# HSPA12B Protects Against Age‐Related Endothelial Cell Senescence by Regulating STING Degradation

**DOI:** 10.1111/acel.70260

**Published:** 2025-10-08

**Authors:** Tingting Li, Peilin Zhu, Joseph Adams, Fei Tu, Jialing Wang, Chloe Garbe, Suman Dalal, Krishna Singh, Xiaojin Zhang, Li Liu, David L. Williams, Chuanfu Li, Xiaohui Wang

**Affiliations:** ^1^ Department of Biomedical Sciences, Quillen College of Medicine East Tennessee State University Johnson City Tennessee USA; ^2^ UMPC Hillman Cancer Center University of Pittsburgh Pittsburgh Pennsylvania USA; ^3^ Health Sciences East Tennessee State University Johnson City Tennessee USA; ^4^ Department of Geriatrics, Jiangsu Provincial Key Laboratory of Geriatrics The First Affiliated Hospital of Nanjing Medical University Nanjing China; ^5^ Department of Surgery, Quillen College of Medicine East Tennessee State University Johnson City Tennessee USA; ^6^ Center of Excellence in Inflammation, Infectious Disease, and Immunity, Quillen College of Medicine East Tennessee State University Johnson City Tennessee USA

## Abstract

Cardiovascular diseases remain the leading cause of mortality worldwide, with aging as a major risk factor. Endothelial cell (EC) dysfunction, driven by cellular senescence, is central to age‐related cardiomyopathy. Despite its clinical significance, the molecular mechanisms underlying endothelial senescence remain incompletely defined. In this study, we observed that the expression of the endothelial‐specific gene heat shock protein family A member 12B (HSPA12B) declines significantly with age. HSPA12B deficiency in mice accelerates age‐related EC senescence and cardiac dysfunction, whereas HSPA12B overexpression mitigates EC senescence, highlighting its protective role against vascular aging. Mechanistically, HSPA12B deficiency impairs X‐box binding protein 1 (XBP1) transcriptional activity and consequently reduces the expression of its downstream target genes suppressor/enhancer of lin‐12‐like (SEL1L) and HMG‐CoA reductase degradation protein‐1 (HRD1). This disruption compromises endoplasmic reticulum‐associated degradation (ERAD) of Stimulator of interferon genes (STING), resulting in persistent activation of the cyclic GMP‐AMP synthase (cGAS)‐STING pathway, a critical driver of EC senescence. In contrast, increased HSPA12B expression enhances XBP1 nuclear translocation and upregulates SEL1L and HRD1, thereby attenuating age‐related STING activation. Importantly, pharmacological inhibition of STING reversed the senescent phenotype caused by HSPA12B deficiency. Similarly, enhancing XBP1 activity restored SEL1L and HRD1 expression, reduced STING activation, and alleviated EC senescence. Conversely, SEL1L deficiency or HRD1 inhibition exacerbated STING activation and abolished the protective effects of HSPA12B. Collectively, these findings reveal a previously unrecognized role for HSPA12B in preserving endothelial homeostasis during aging by regulating XBP1‐mediated ER‐associated degradation of STING and highlight HSPA12B as a potential therapeutic target for age‐related cardiovascular dysfunction.

## Graphical abstract

Activation of the cGAS–STING pathway contributes to age‐related cardiac endothelial senescence, fibrotic remodeling, and functional decline. HSPA12B preserves endothelial homeostasis during aging by promoting XBP1‐dependent, SEL1L/HRD1‐mediated endoplasmic reticulum–associated degradation of STING, thereby attenuating cardiac endothelial senescence.

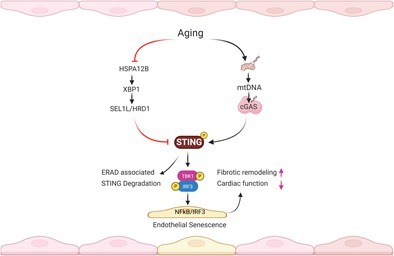

## 1 Introduction

Age is a major risk factor for cardiovascular‐associated diseases (CVD), which remain the leading cause of death worldwide (Booth et al. 2023; Li et al. 2020; Martin et al. 2025). In the United States, the population aged 65 and over has reached 58 million in 2022 and is expected to reach 82 million by 2050 (Bureau 2023). Notably, approximately 80% of all cardiovascular deaths occur in individuals over the age of 65 (Sidney et al. 2019). Despite significant advances in therapy interventions, CVD‐related mortality continues to increase, driven largely by the expanding aging population. Therefore, there is an urgent need to develop novel strategies to improve cardiac functional recovery, attenuate cardiac remodeling, and prevent heart failure in elderly patients. A better understanding of the molecular mechanisms underlying aging‐related cardiac remodeling and dysfunction is essential to develop effective treatments.

Endothelial cells (ECs), which collectively form the largest organ system in the body, are essential for maintaining vascular integrity, immune regulation, and metabolic homeostasis (Reiterer and Branco 2020). However, endothelial cell dysfunction is a hallmark of aging and a key contributor to the development of age‐related cardiovascular diseases (Brandes et al. 2005; Sayed et al. 2021; Ungvari et al. 2018). A key feature of endothelial aging is the accumulation of senescent cells, characterized by cell cycle arrest, altered gene expression, and the secretion of senescence‐associated secretory phenotype (SASP) factors (Bloom et al. 2023; Gevaert et al. 2017; Hemanthakumar et al. 2021; Minamino et al. 2002; Yin et al. 2022). These SASP factors, which include pro‐inflammatory cytokines, chemokines, and matrix metalloproteinases, have deleterious paracrine effects on surrounding tissues and drive the progression of multiple cardiovascular pathogenesis (Gevaert et al. 2017; Yin et al. 2022). Co‐localization studies of senescence markers with the endothelial marker CD31 confirm that endothelial cells are the predominant senescent population within the aged myocardium. Moreover, analysis of publicly available single‐cell RNA sequencing datasets from aged cardiac tissues further supports that endothelial cells are the major senescent cell type in the heart. Despite these observations, the molecular mechanisms driving endothelial senescence in the context of cardiovascular aging remain incompletely understood.

The cGAS‐STING pathway is a crucial sensor of cytosolic DNA and a central regulator of innate immunity (Dvorkin et al. 2024; Sun and Hornung 2022). Under physiological conditions, cGAS–STING signaling is tightly controlled. However, persistent activation of this pathway, driven by cytoplasmic double‐stranded DNA accumulation from mitochondrial dysfunction or genomic instability, is a major driver of cellular senescence and tissue decline during aging (Jiménez‐Loygorri et al. 2024; Yu et al. 2022; Zhao et al. 2023; Zhong et al. 2022). Although cGAS–STING signaling has been implicated in aging‐related pathologies, the mechanisms that restrain or resolve this pathway remain poorly defined.

HSPA12B, an endothelial‐specific member of the HSP70 family, has previously been shown to regulate angiogenesis, particularly in ischemia–reperfusion injury models (Han et al. 2003; Li et al. 2013; Steagall et al. 2006; Tu et al. 2020; Zhang et al. 2020). However, its role in age‐related endothelial dysfunction and senescence has not been explored. In this study, we demonstrate that HSPA12B expression significantly declines with aging. Loss of HSPA12B exacerbates endothelial senescence and cardiac dysfunction, while HSPA12B overexpression rejuvenates aged ECs. Intriguingly, HSPA12B deficiency exacerbates cGAS–STING signaling activation during aging. Mechanistically, we show that HSPA12B interacts with XBP1, promoting its nuclear translocation and transcriptional activation of ERAD genes, including SEL1L and HRD1. SEL1L is essential for targeting STING for proteasomal degradation (Ji et al. 2023). HSPA12B deficiency disrupts this regulatory axis, leading to impaired ERAD function and persistent STING activation. Furthermore, genetic or pharmacologic enhancement of XBP1 or SEL1L expression attenuates STING activation and endothelial senescence, whereas inhibition of this pathway abolishes the protective effects of HSPA12B.

Collectively, our findings identify HSPA12B as a critical regulator of endothelial homeostasis and vascular aging. By facilitating ERAD‐mediated STING degradation, HSPA12B protects against age‐related endothelial senescence and cardiovascular dysfunction.

## 2 Results

### 2.1 Endothelial Cells Are the Predominant Senescent Cell Type in the Aged Heart

**FIGURE 1:**
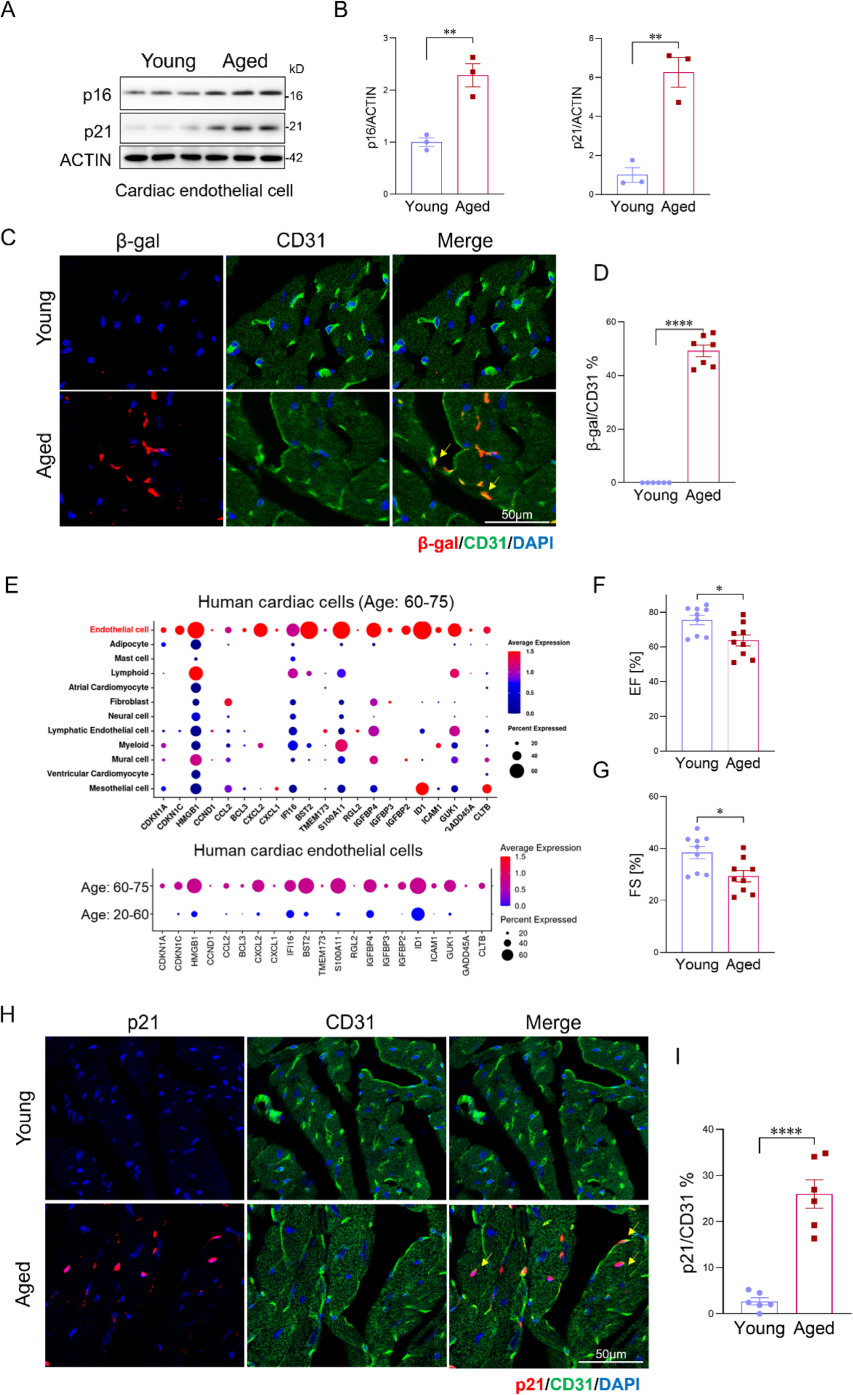
Endothelial cells are the predominant senescent cell type in the aged heart. (A) Western blot analysis of senescence markers p16 and p21 in cardiac endothelial cells isolated from young (3–5 months) and aged (22–24 months) mice (*n* = 6 mice/group). (B) Quantification of the Western blot shown in (A), normalized to ACTIN. (C) Representative confocal immunofluorescent images of β‐gal (red) co‐stained with the endothelial marker CD31 (green) in heart sections from young and aged mice. Nuclei were counterstained with DAPI (blue). Scale bar, 50 μm. (D) Quantification of β‐gal–positive endothelial cells (% of CD31^+^ cells) in young and aged mouse hearts (*n* = 6 mice/group). (E) Analysis of publicly available single‐cell RNA sequencing (scRNA‐seq) data from human hearts of young (20–60 years) and elderly (60–75 years) individuals (ERP123138). (F, G) Echocardiographic analysis revealed impaired cardiac function in aged mice, with reduced ejection fraction (EF) and fractional shortening (FS) compared to young controls (*n* = 9 mice/group). (H, I) Immunofluorescent co‐staining and quantification for p21 (red) and the endothelial marker CD31 (green) in heart sections from young and aged mice (*n* = 6 mice/group). Scale bars, 50 μm. Data are presented as the mean ± SD. *p < 0.05, **p < 0.01, ***p < 0.001. Ef, ejection fraction; FS, fractional shortening; scRNA‐seq, single‐cell RNA sequencing.

Cellular senescence has emerged as a key contributor to the pathogenesis of age‐related cardiomyopathy (Childs et al. 2018; Evangelou et al. 2023; Gevaert et al. 2017; Hemanthakumar et al. 2021; Marino et al. 2022). To assess cellular senescence in the aging heart, we evaluated the expression of senescence marker expression in cardiac endothelial cells isolated from young (3–5 m) and aged (22–24 m) mice, and measured key components of the SASP, including chemokine (C‐X‐C motif) ligand (CXCL)2, CXCL9, Interleukin‐1 alpha (IL‐1A), and tumor Necrosis Factor Alpha (TNFα) of young and aged mouse heart tissues (Figure 1A,B and Figure S1A–C). Aged mouse hearts exhibited a significant increase in senescence‐associated β‐galactosidase (SA‐β‐gal) positive staining and elevated expression of senescence and SASP markers, accompanied by enhanced fibrotic remodeling (Figure 1C,D and Figure S1D,E) and impaired cardiac function, as evidenced by reduced ejection fraction (EF) and fractional shortening (FS) compared with young controls (Figure 1F,G). To further define the identity of senescent cell populations, we analyzed publicly available single‐cell RNA sequencing (scRNA‐seq) data from human cardiac tissues of elderly individuals (ages 60–75 years) (Kanemaru et al. 2025). scRNA‐seq analysis revealed that cardiac endothelial cells exhibited significantly higher expression of senescence‐associated genes compared with other cardiac cell types (Figure 1E). Additionally, we compared the senescence signature of cardiac endothelial cells from young (20–60 years) and aged (60–75 years) individuals. Compared with young, cardiac endothelial cells from aged individuals showed increased expression of senescence‐related genes. To validate these findings in mice, we performed immunofluorescent co‐staining of the senescence markers cyclin‐dependent kinase inhibitor 1A (Cdkn1a/p21) and cyclin‐dependent kinase inhibitor 2A (Cdkn2a/p16) alongside the endothelial marker CD31 in heart tissues from young (3–5 months) and aged (22–24 months) mice. As expected, the majority of p21‐ and p16‐positive cells in aged hearts co‐localized with CD31, confirming that endothelial cells represent the predominant senescent population in the aging heart (Figure 1H,I, Figure S1F,G). Collectively, these results indicate that endothelial cells represent the predominant senescent cell population in the aged heart.

### 2.2 Endothelial Cell HSPA12B Deficiency Leads to Age‐Related Cardiac Endothelial Cell Senescence and Cardiomyopathy

**FIGURE 2:**
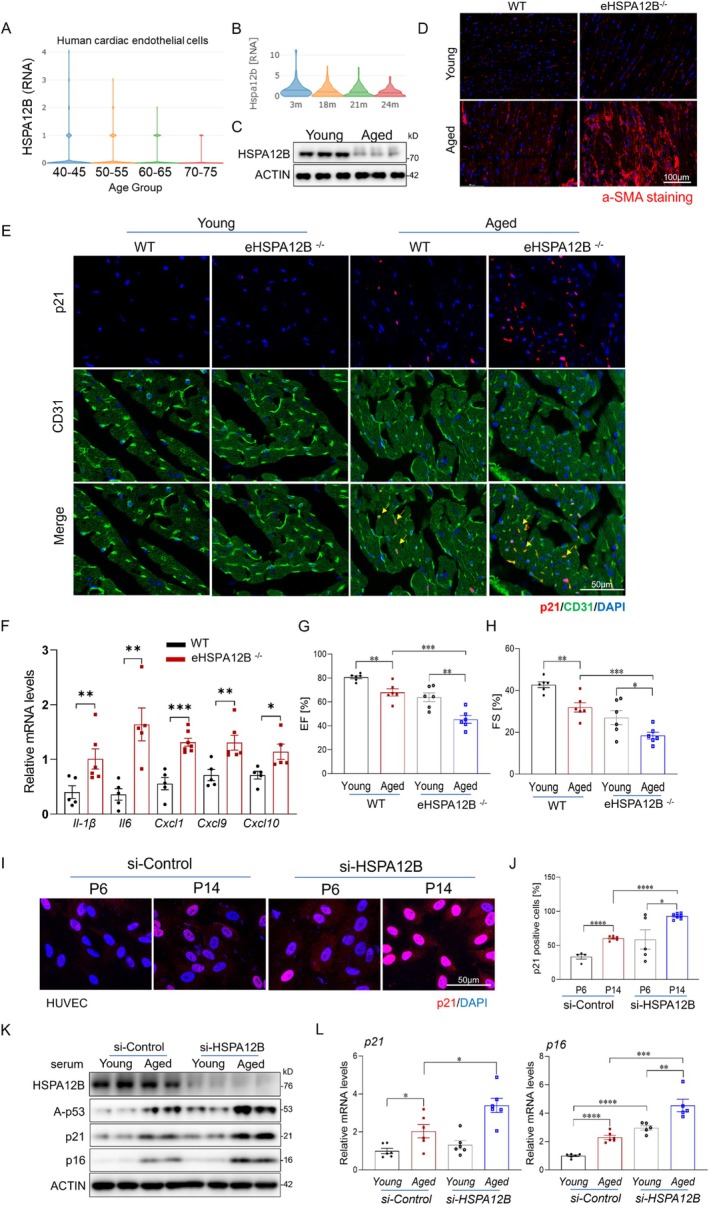
HSPA12B deficiency promotes age‐related cardiac endothelial cell senescence. (A, B) Analysis of publicly available single‐cell RNA sequencing datasets of cardiac endothelial cells from aged humans and mice compared with younger counterparts. (C) Western blot analysis of HSPA12B expression in cardiac endothelial cells isolated from aged versus young mice (*n* = 3 mice/group). (D) Immunofluorescent staining for α‐smooth muscle Actin (α‐SMA) in heart sections from 16‐month‐old endothelial‐specific HSPA12B‐deficient (eHSPA12B^−/−^) mice and wild‐type (WT) controls. Scale bars, 100 μm. (E) Immunofluorescence staining of p21 in heart sections from 16‐month‐old WT and eHSPA12B^−/−^ mice. Scale bars, 50 μm. (F) Quantitative PCR analysis of SASP factors (IL‐1β, IL‐6, CXCL1, CXCL9, and CXCL10) in heart tissue from aged eHSPA12B^−/−^ and WT mice (*n* = 6 mice/group). (G, H) Echocardiographic measurements of ejection fraction (EF) and fractional shortening (FS) in aged eHSPA12B^−/−^ and WT mice (*n* = 6 mice/group). (I, J) Representative images and quantification of p21‐positive endothelial cells in the indicated groups. Scale bars, 50 μm. (*n* = 6/group). (K) Western blot analysis of p16, p21, and acetylated p53 in HUVECs transfected with si‐Control or si‐HSPA12B and treated with from young (4–6 months) or aged (20–24 months) mice for 24 h (*n* = 4/group). (L) qRT‐PCR analysis of p16 and p21 mRNA expression levels in the indicated groups (*n* = 6/group). Data are presented as the mean ± SD. *p < 0.05, **p < 0.01, ***p < 0.001. α‐SMA, α‐smooth muscle Actin; EF, ejection fraction; FS, fractional shortening; HUVECs, human umbilical vein endothelial cells; SASP, senescence‐associated secretory phenotype.

HSPA12B is a member of the HSP70 family of proteins that is predominantly expressed in endothelial cells (Steagall et al. 2006). Our previous studies have shown that endothelial cell HSPA12B plays a critical role in regulating angiogenesis and modulating immune responses (Tu et al. 2020; Zhang et al. 2020). To explore the potential role of HSPA12B in cardiac aging, we analyzed publicly available single‐cell RNA sequencing datasets and observed a significant reduction in HSPA12B expression in cardiac endothelial cells from aged humans and mice (Figure 2A,B) (Litviňuková et al. 2020). Consistently, cardiac endothelial cells isolated from aged mice also exhibited markedly lower HSPA12B expression compared to those from young mice (Figure 2C). To determine whether reduced endothelial HSPA12B expression contributes to age‐related cardiac dysfunction, we generated endothelial‐specific HSPA12B‐deficient (eHSPA12B^−/−^) mice and assessed their cardiac phenotype during aging (Figure S2A). Remarkably, by 16 months of age, eHSPA12B^−/−^ mice exhibited pronounced cardiac fibrotic remodeling and significant functional decline compared to age‐matched wild‐type (WT) controls (Figure 2D,G,H and Figure S2B). Notably, these pathological features typically appeared in WT mice only at 24 months of age or older, suggesting that HSPA12B deficiency accelerates cardiac aging.

We next evaluated whether HSPA12B deficiency contributes to age‐related endothelial senescence. Hearts from eHSPA12B^−/−^ mice showed significantly increased endothelial cell senescence as indicated by elevated expression of the senescence markers p21, p16, and β‐gal (Figure 2E and Figure S2C,D), along with increased levels of SASP cytokines and chemokines compared to WT controls (Figure 2F). To further define the role of HSPA12B in endothelial senescence, we transfected human umbilical vein endothelial cells (HUVECs) with control siRNA (siRNA‐Con) or siRNA targeting HSPA12B (siRNA‐HSPA12B) and exposed them to from young (3–5 m) and aged (22–24 m) mice for 24 h. Knockdown of HSPA12B exacerbated aged serum–induced endothelial senescence, as evidenced by increased expression of p16, p21, and acetylated p53, along with enhanced SA‐β‐gal staining (Figure 2K,L, Figure S3D,E). Furthermore, aged HUVECs (P14) transfected with siRNA targeting HSPA12B exhibited accelerated replicative senescence, further supporting the link between reduced HSPA12B expression and enhanced endothelial cell senescence (Figure 2I,J and Figure S3A–C). Collectively, these findings demonstrate that reduced HSPA12B expression exacerbates endothelial cell senescence, which contributes to age‐related cardiac dysfunction.

### 2.3 HSPA12B Deficiency Exacerbates Aging Associated Activation of the cGAS‐STING Pathway

**FIGURE 3:**
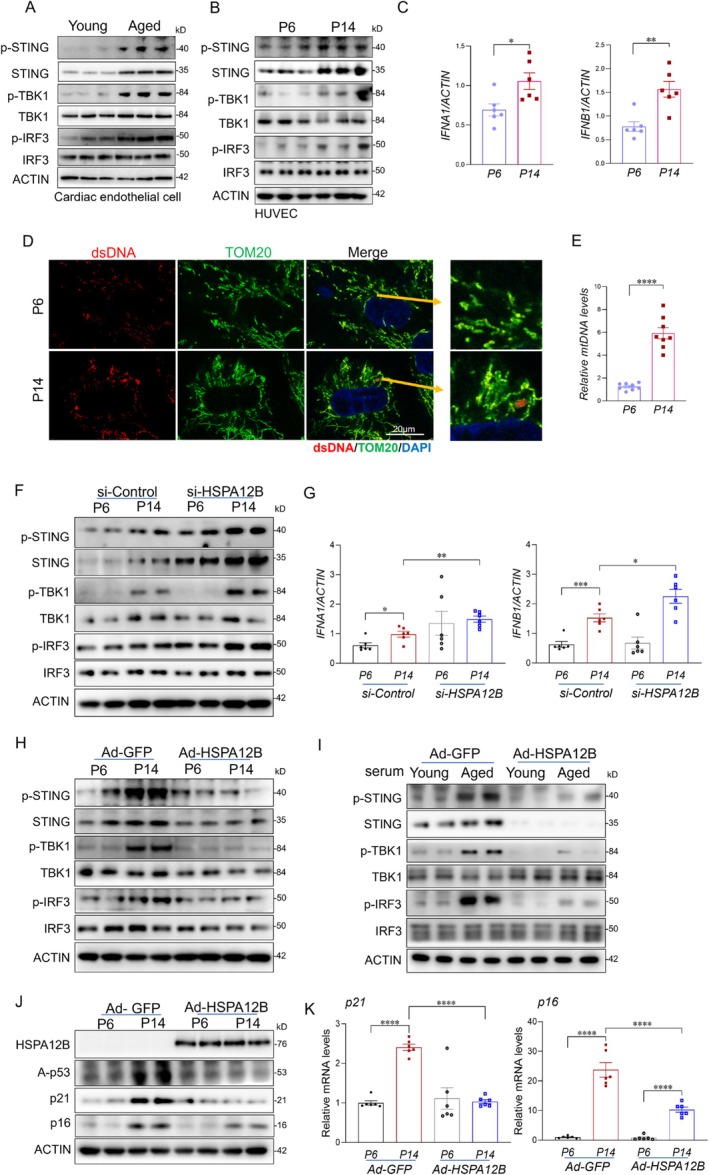
HSPA12B Modulates cGAS–STING pathway activation and endothelial senescence during aging. (A) Western blot analysis of STING pathway components in cardiac endothelial cells isolated from young and aged mice (*n* = 3 mice/group). (B) Western blot analysis of phosphorylated STING, TBK1, IRF3, and IFNA expression in young (P < 6) and aged (P12–16) HUVECs. (C) qRT‐PCR analysis of IFNα and IFNβ relative to ACTIN expression levels in the indicated groups (*n* = 6/group). (D) Immunofluorescent staining of cytosolic double‐stranded DNA (dsDNA) in young and aged HUVECs. Scale bar: 20 μm. (E) Quantification of cytosolic mtDNA by qPCR in young and aged HUVECs (*n* = 6/group). (F) Western blot analysis of STING pathway activation in aged HUVECs transfected with si‐Control or si‐HSPA12B. (G) qRT‐PCR analysis of relative IFNα and IFNβ expression levels in the indicated groups (*n* = 6/group). (H) Western blot analysis of STING pathway components in young and aged HUVECs transduced with adenoviruses encoding HSPA12B (Ad‐HSPA12B) or GFP (Ad‐GFP). (I) Western blot analysis of STING pathway components in young and aged HUVECs transduced with Ad‐HSPA12B or Ad‐GFP treated with aged mouse serum. (J) Western blot analysis of senescence markers in the indicated groups. (K) qRT‐PCR analysis of senescence markers in the indicated HUVEC groups. Data are presented as the mean ± SD. *p < 0.05, **p < 0.01, ***p < 0.001. DsDNA, double‐stranded DNA; mtDNA, mitochondrial DNA.

The cGAS–STING signaling pathway plays a central role in sensing cytosolic DNA and driving cellular senescence, particularly in the context of aging (Glück et al. 2017; Guo et al. 2021; Liu et al. 2023). We first examined the activation status of this pathway in cardiac endothelial cells isolated from young and aged mice, as well as in young (passage <P6) and aged (passages P12–16) HUVECs. Heart tissues and endothelial cells from aged mice exhibited increased phosphorylation of STING, TANK‐binding kinase 1 (TBK1), and Interferon regulatory factor 3 (IRF3) compared to young controls, indicating activation of the pathway (Figure 3A and Figure S4A–C). Similarly, aged HUVECs showed elevated levels of phosphorylated STING, TBK1, and IRF3, along with increased expression of downstream target genes, Interferon alpha and beta (IFNα and IFNβ) (Figure 3B,C and Figure S4D).

Mitochondrial DNA (mtDNA) is a key activator of cGAS–STING signaling, and mitochondrial dysfunction is a well‐recognized hallmark of aging (He et al. 2022; Jiménez‐Loygorri et al. 2024; Zhong et al. 2022). To assess whether cytosolic accumulation of mtDNA occurs in aged endothelial cells, we quantified cytosolic DNA levels in young and aged HUVECs using quantitative PCR. Compared to young cells, aged HUVECs exhibited a significant increase in cytosolic mtDNA (Figure 3E). Consistently, immunofluorescent staining for double‐stranded DNA (dsDNA) revealed a marked accumulation of cytosolic dsDNA in aged HUVECs (Figure 3D), supporting enhanced mtDNA leakage into the cytosol during aging. We further evaluated cGAS expression in endothelial cells. Aged HUVECs showed significantly increased levels of cGAS compared with young cells (Figure S4E). However, modulation of HSPA12B (via Ad‐HSPA12B overexpression or si‐HSPA12B knockdown) did not significantly alter cGAS expression (Figure S4F,G). In contrast, HSPA12B deficiency specifically enhanced downstream signaling, as evidenced by increased levels of both total and phosphorylated STING, phosphorylation of TBK1, and IRF3, and upregulation of IFNα and IFNβ expression (Figure 3F,G). Together, these findings demonstrate that reduced HSPA12B expression exacerbates aging‐related cGAS–STING pathway activation in endothelial cells and likely contributes to the enhanced senescence phenotype observed during vascular aging.

### 2.4 Increased Expression of HSPA12B Attenuates Age‐Related cGAS‐STING Activation and Endothelial Senescence

To further investigate the role of HSPA12B in regulating age‐associated cGAS–STING activation and endothelial senescence, we increased HSPA12B expression using an adenoviral vector (Ad‐HSPA12B) in replicative‐aged HUVECs (Figure S3F). Ad‐GFP was used as a control. In parallel, young HUVECs were transduced with Ad‐HSPA12B for 24 h and subsequently exposed to aged mouse serum to mimic systemic aging. As shown in Figure 3H,I, increased expression of HSPA12B significantly attenuated activation of the cGAS–STING pathway under both replicative aging and aged serum conditions. This was evidenced by reduced phosphorylation of STING, TBK1, and IRF3 compared to control groups.

We next examined whether HSPA12B overexpression could mitigate endothelial senescence in the context of replicative aging and exposure to aged serum. As shown in Figure 3, increased HSPA12B expression significantly reduced the expression of key senescence markers, including p16, p21, and acetylated p53 (Figure 3J,K and Figure S3J,K). Furthermore, SA‐β‐galactosidase staining was decreased in cells with increased expression of HSPA12B compared to GFP controls (Figure S3G–J). To further investigate the role of STING activation in HSPA12B deficiency‐induced endothelial cell senescence, we inhibited STING activation with C‐176 in HUVECs with and without HSPA12B knockdown by its specific siRNA. As shown in Figure S4H–I, HSPA12B knockdown led to increases in both total and phosphorylated STING, as well as phosphorylation of TBK1 and IRF3. These changes were accompanied by elevated levels of senescence markers p16 and p21. Inhibition of STING activation reduced this pathway activation and attenuated cellular senescence caused by HSPA12B knockdown. Together, these findings suggest that HSPA12B exerts protective effects against age‐related STING activation, which contributes to endothelial senescence.

### 2.5 STING Inhibition Attenuates Cardiac Endothelial Senescence in Aged Wild‐Type and HSPA12B Deficient Mice

**FIGURE 4:**
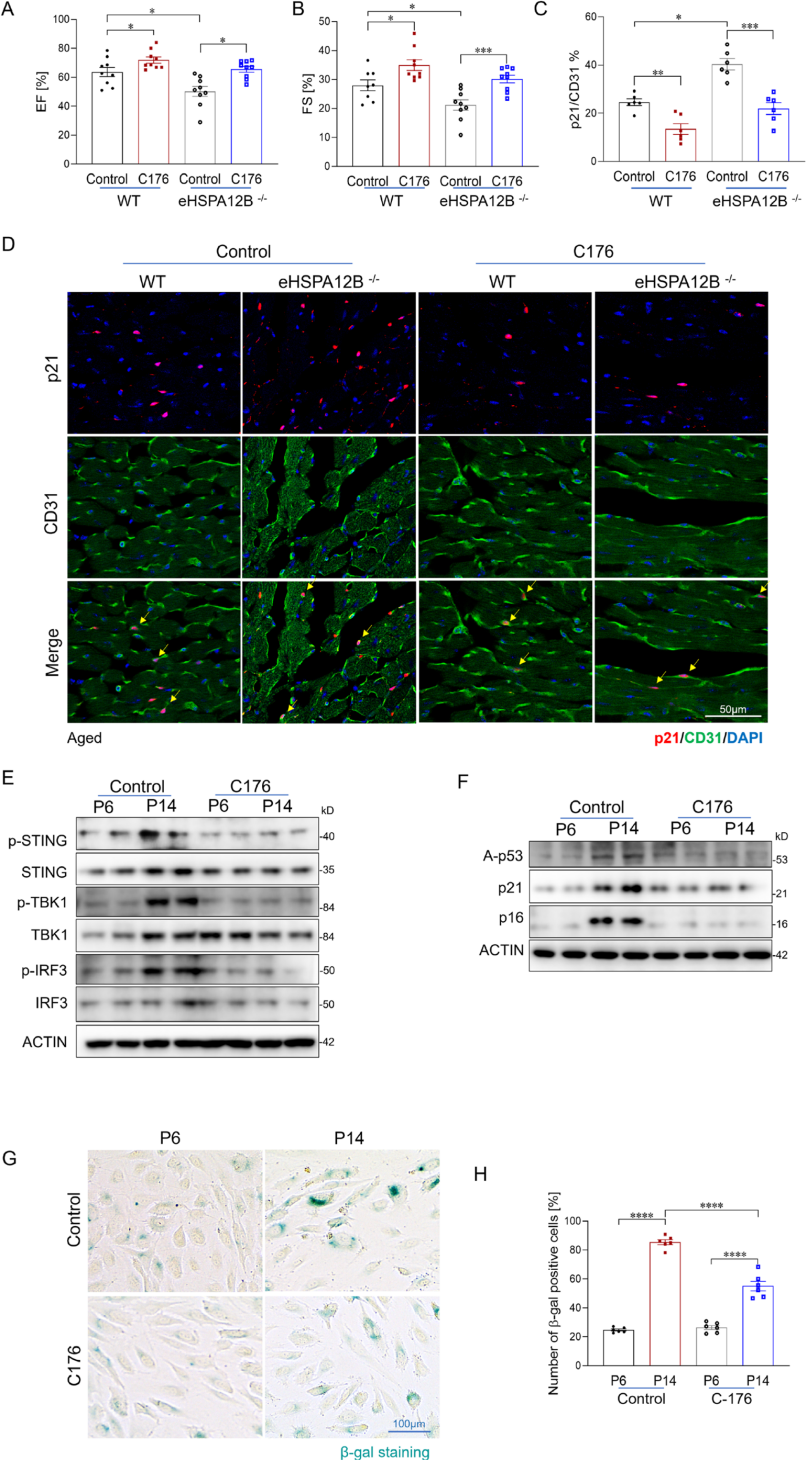
STING inhibition attenuates cardiac endothelial senescence in aged wild‐type and HSPA12B deficient mice. (A, B) Echocardiographic assessment of EF and FS in aged WT and eHSPA12B^−/−^ mice treated with the STING inhibitor C‐176 or vehicle every other day for 1 month (*n* = 6–9 mice/group). (C‐D) Quantification and immunofluorescent co‐staining of CD31 and p21 in heart tissues from C‐176– and vehicle‐treated aged WT and eHSPA12B^−/−^ mice (*n* = 6/group). Scale bars, 50 μm. (E) Western blot analysis of phosphorylated STING, TBK1, and IRF3 in young (P6) and aged (P14) HUVECs treated with C‐176. (F) Expression of senescence markers of the same HUVEC groups. (G, H) Representative images and quantification of β‐galactosidase staining of aged HUVECs treated with C‐176 or vehicle. Scale bars, 100 μm. Data are presented as the mean ± SD. *p < 0.05, **p < 0.01, ***p < 0.001. EF, ejection fraction; FS, fractional shortening.

We then asked whether reducing STING activation could rescue age‐related endothelial senescence and cardiac dysfunction caused by HSPA12B deficiency in vivo. To test this, we treated aged WT and eHspa12b^−/−^ mice with the STING inhibitor C‐176 (Chen et al. 2025) every other day for 1 month. Cardiac function was assessed via echocardiography. C‐176 treatment significantly improved ejection fraction (EF) and fractional shortening (FS) in both aged WT and eHSPA12B^−/−^ mice compared with vehicle‐treated controls (Figure 4A,B).

To further confirm whether C‐176 effectively inhibits STING pathway activation in cardiac endothelial cells, we performed immunofluorescent co‐staining of phosphorylated STING (p‐STING), TBK1 (p‐TBK1), and IRF3 (p‐IRF3) with the endothelial marker CD31. As shown in Figure S5, compared with aged WT mice, eHSPA12B^−/−^ mice displayed strong activation of the STING pathway within cardiac endothelial cells, as indicated by increased phosphorylation of STING, TBK1, as well as elevated nuclear levels of p‐IRF3. However, C‐176 treatment significantly attenuated this activation in cardiac endothelial cells in both aged WT and eHSPA12B^−/−^ mice.

To assess whether STING inhibition reduces age‐related cardiac endothelial senescence in vivo, we performed immunofluorescent co‐staining of the endothelial marker CD31 with the senescence markers p21 and p16. As shown in Figure 4C,D and Figure S6A,B, C176 treatment markedly reduced the number of senescent endothelial cells in both aged (22–24 months) WT and eHSPA12B^−/−^ mice, indicating that STING inhibition alleviates endothelial senescence in aged mice.

To further determine whether STING activation directly contributes to endothelial senescence, HUVECs were treat with C‐176 in the context of replicative aging and aged serum exposure. In aged HUVECs, C‐176 treatment significantly reduced the expression of senescence markers p16, p21, and acetylated p53 (Figure 4E,F and Figure S6E,F). SA‐β‐galactosidase staining also revealed a substantial reduction in senescent cell burden following STING inhibition (Figure 4G,H and Figure S6C,D). Furthermore, quantitative PCR analysis demonstrated downregulation of key SASP‐associated cytokines and chemokines, further supporting the anti‐senescent effect of STING inhibition in aged endothelial cells (Figure S6G).

Together, these results demonstrate that inhibition of STING alleviates age‐related cardiac endothelial senescence and preserves cardiac function in both WT and HSPA12B‐deficient mice, highlighting the therapeutic potential of targeting STING signaling in vascular aging.

### 2.6 HSPA12B Deficiency Impairs ER Associated Degradation of STING

**FIGURE 5:**
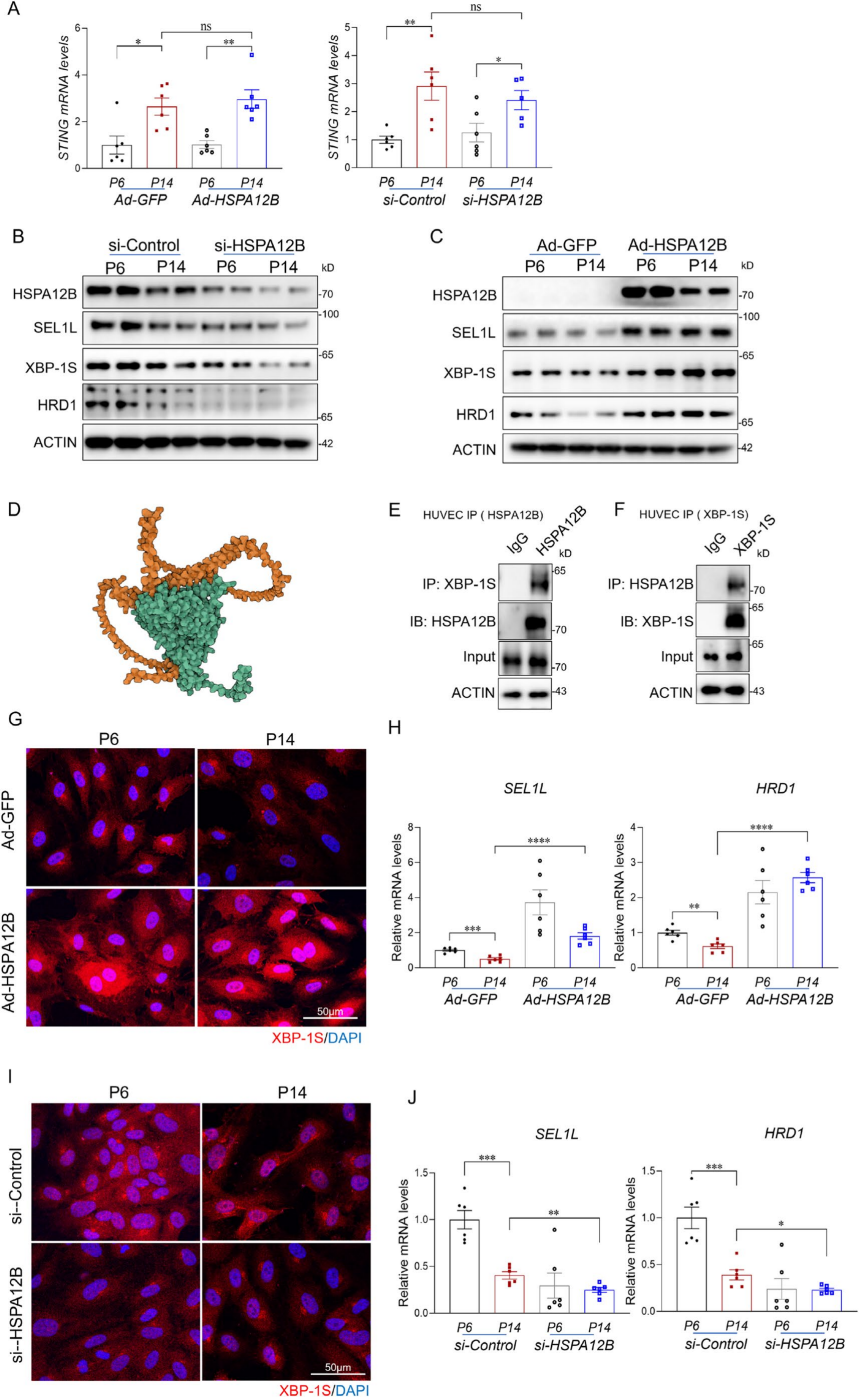
HSPA12B deficiency impairs ER associated degradation of STING. (A) qPCR analysis of STING mRNA levels following HSPA12B knockdown or overexpression in endothelial cells (*n* = 6/group). (B, C) Expression levels of SEL1L and HRD1 in aged endothelial cells with HSPA12B knockdown (B) or overexpression (C). (D) Structural modeling using AlphaFold to predict the potential interaction interface between HSPA12B and XBP1. (E, F) Co‐immunoprecipitation assays to evaluate the interaction between HSPA12B and XBP1 in the indicated conditions. (G) Immunofluorescent staining of XBP1s in young and aged endothelial cells following HSPA12B overexpression. Scale bars, 50 μm. (H) qRT‐PCR analysis of SEL1L and HRD1 expression in the indicated HUVEC groups (*n* = 6/group). (I) Immunofluorescent staining of XBP1s in young and aged endothelial cells under HSPA12B‐deficient and control conditions. Scale bars, 50 μm. (J) qRT‐PCR analysis of SEL1L and HRD1 expression in the indicated groups (*n* = 6/group). Data are presented as the mean ± SD. *p < 0.05, **p < 0.01, ***p < 0.001.

To elucidate the mechanism by which HSPA12B regulates cGAS–STING activation, we first examined STING protein expression in aged mouse hearts and senescent endothelial cells. STING protein levels were significantly elevated in both, and this increase was further augmented by HSPA12B deficiency (Figure 3A,B,F). In contrast, overexpression of HSPA12B significantly reduced STING protein levels (Figure 3H,I). We next investigated whether HSPA12B regulates STING transcriptionally. qPCR analysis revealed no significant differences in STING mRNA levels following either HSPA12B knockdown or overexpression, suggesting that HSPA12B modulates STING expression at a post‐transcriptional level (Figure 5A).

Given that STING protein turnover is regulated by SEL1L/HRD1‐mediated ER‐associated degradation (ERAD) (Ji et al. 2023), we examined whether HSPA12B affects the expression of SEL1L and HRD1. Both SEL1L and HRD1 were downregulated in aged endothelial cells, and their expression was further reduced in cells with HSPA12B knockdown (Figure 5B,J). In contrast, HSPA12B overexpression significantly upregulated SEL1L and HRD1 expression (Figure 5C,H).

As SEL1L and HRD1 are transcriptional targets of XBP1 (Kaneko et al. 2007; Yamamoto et al. 2008), we hypothesized that HSPA12B may influence XBP1 activity. Structural modeling using AlphaFold predicted a direct interaction between HSPA12B and XBP1, which we validated by co‐immunoprecipitation, confirming a physical interaction (Figure 5D,G,I).

These results suggest that HSPA12B enhances XBP1 transcriptional activity, thereby upregulating SEL1L and HRD1 expression to promote ERAD‐mediated degradation of STING. This regulatory mechanism reveals a previously unrecognized role for HSPA12B in limiting aberrant cGAS–STING signaling activation and preserving endothelial homeostasis during aging.

### 2.7 ERAD Inhibition Diminishes the Protective Effects of HSPA12B on cGAS‐STING Activation and Endothelial Senescence

**FIGURE 6:**
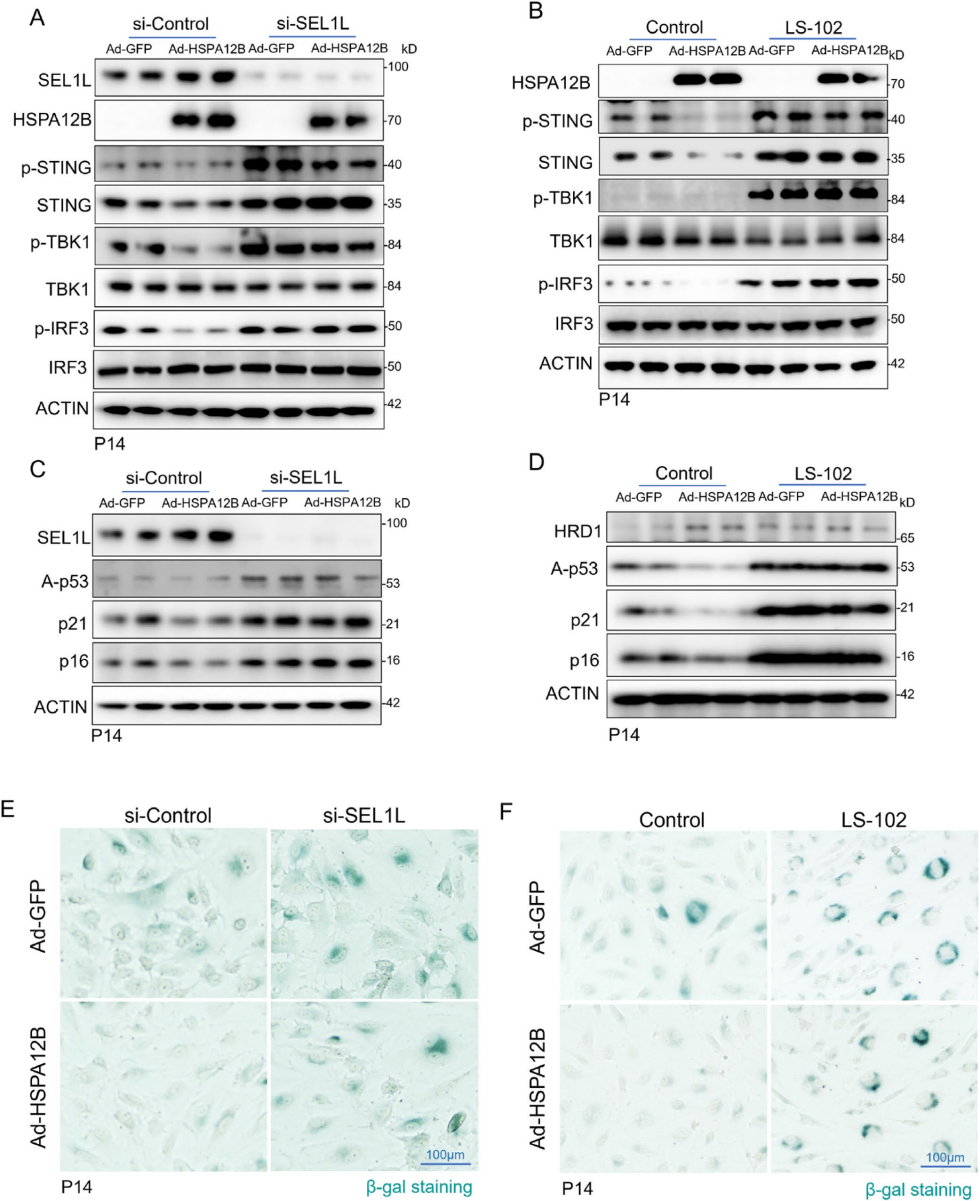
ERAD inhibition diminishes the protective effects of HSPA12B on cGAS‐STING activation and endothelial senescence. (A) Aged HUVECs were transfected with siRNA targeting SEL1L or control siRNA, followed by adenoviral overexpression of HSPA12B (Ad‐HSPA12B) or GFP (Ad‐GFP). Activation of the STING pathway was assessed by immunoblotting. (B) Aged HUVECs were treated with the HRD1 inhibitor LS‐102 to inhibit ERAD, followed by Ad‐HSPA12B or Ad‐GFP transduction, and analyzed for STING protein levels and pathway activation. (C, D) Immunoblot analysis of senescence markers in aged HUVECs following ERAD disruption and HSPA12B overexpression. (E, F) Representative β‐gal staining in the indicated groups. Scale bars, 100 μm.

To further validate the role of ERAD‐mediated STING degradation in the protective effects of HSPA12B against age‐related cGAS–STING activation and endothelial senescence, we performed both genetic (siRNA‐mediated) and pharmacological inhibition of ERAD in aged endothelial cells. We first silenced SEL1L, an adaptor protein for the ERAD E3 ubiquitin ligase Hrd1 (Ji et al. 2023; Lin et al. 2024), in aged HUVECs using siRNA, followed by transduction with either Ad‐HSPA12B or Ad‐GFP as a control. As shown in Figures 3I and 5D, HSPA12B overexpression increased SEL1L expression and attenuated cGAS–STING activation, as evidenced by decreased phosphorylation of STING, TBK1, and IRF3, compared to the Ad‐GFP control group. However, knockdown of SEL1L abolished the ability of HSPA12B to suppress cGAS‐STING signaling, leading to a significant increase in STING expression and phosphorylation (Figure 6A). These results suggest that SEL1L is essential for HSPA12B‐mediated STING degradation.

Consistent with these findings, pharmacological inhibition of ERAD using the HRD1 inhibitor LS‐102 (Fujita et al. 2015) in aged HUVECs led to a significant increase in STING protein levels and abolished the suppressive effects of HSPA12B on cGAS–STING pathway activation (Figure 6B). Importantly, both SEL1L knockdown and ERAD inhibition reversed the anti‐senescent effects of HSPA12B in aged HUVECs. Specifically, we observed increased expression of senescence markers p16, p21, and acetylated p53, enhanced SA‐β‐galactosidase activity, and elevated p21‐positive staining in cells treated with ERAD‐disrupting interventions despite HSPA12B overexpression (Figure 6C–F, Figures S7A–D and S8A,B). Together, these findings indicate that ERAD‐mediated degradation of STING is critical for the protective effects of HSPA12B against aberrant STING activation and endothelial senescence during aging.

### 2.8 XBP1 Regulates Age‐Related cGAS–STING Activation and Endothelial Senescence

**FIGURE 7:**
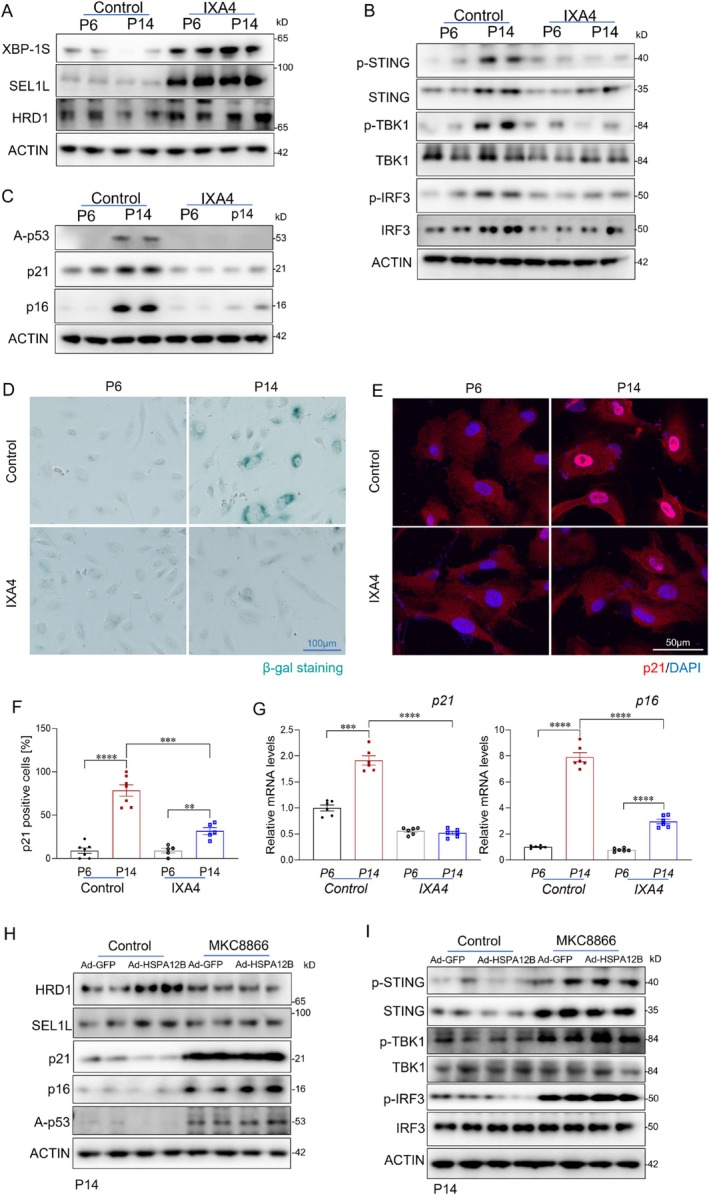
XBP1 regulates Age‐Related cGAS–STING Activation and Endothelial Senescence. (A‐B) Young (P ≤ 6) and aged (P12–16) HUVECs were treated with the XBP1 activator IXA4 or vehicle control and analyzed for expression of SEL1L, HRD1, and phosphorylation of STING, TBK1, and IRF3. (C) Immunoblot analysis of senescence markers following IXA4 treatment in aged HUVECs (*n* = 4/group). (D) Representative β‐gal staining in the indicated groups. Scale bars, 100 μm. (E) Immunofluorescent staining of p21 in the indicated groups. Scale bars, 50 μm. (F) Quantification of p21‐positive endothelial cells in the indicated groups (*n* = 6/group). (G) qPCR analysis of senescence markers in the indicated groups (*n* = 6/group). (H) Immunoblot analysis of senescence markers in aged HUVECs treated with MKC8866, followed by adenoviral overexpression of HSPA12B (Ad‐HSPA12B) or GFP (Ad‐GFP) (*n* = 4/group). (I) Immunoblot analysis of senescence markers in the indicated groups (*n* = 4/group). Data are presented as the mean ± SD. *p < 0.05, **p < 0.01, ***p < 0.001.

XBP1 is a key regulator of ER‐associated degradation (ERAD) by promoting the expression of SEL1L and HRD1 (Kaneko et al. 2007; Yamamoto et al. 2008). To investigate the role of XBP1 in age‐related cGAS–STING activation and endothelial senescence, aged HUVECs were treated with the XBP1 activator IXA4 (Cabral‐Miranda et al. 2022; Sheng et al. 2019) (Figure S8C,D). IXA4 treatment significantly upregulated SEL1L and HRD1 expression and attenuated cGAS–STING activation, as indicated by decreased phosphorylation of STING, TBK1, and IRF3 (Figure 7A,B and Figure S8F). Additionally, IXA4 markedly attenuated endothelial senescence, as demonstrated by decreased levels of p16, p21, and acetylated p53 (Figure 7C,D), as well as reduced p21 immunostaining and SA‐β‐galactosidase activity compared with vehicle‐treated controls (Figure 7E–G).

To further determine whether XBP1 is required for the protective effects of HSPA12B, aged HUVECs were treated with the XBP1 inhibitor MKC8866 (Sheng et al. 2019) following Ad‐HSPA12B transduction. Inhibition of XBP1 abrogated the HSPA12B mediated upregulation of SEL1L and HRD1 and abolished its suppressive effects on STING activation (Figure 7H,I). Furthermore, XBP1 inhibition negated the anti‐senescent effects of HSPA12B, leading to increased expression of p16, p21, and acetylated p53, as well as elevated p21 staining and SA‐β‐gal positivity (Figure 7H, Figure S8E,G–I). These findings indicate that XBP1 plays a critical role in mediating the protective effects of HSPA12B on age‐associated cGAS–STING activation and endothelial senescence and highlight XBP1 as an important regulator of the cGAS–STING signaling and vascular aging.

## 3 Discussion

Cellular senescence has been increasingly recognized as a key driver of age‐related cardiac functional decline (Bloom et al. 2023; Culley and Chan 2022; Gevaert et al. 2017; Minamino et al. 2002). However, the identity of the predominant senescent cell types within the aged heart and the mechanisms governing their senescence remain incompletely defined. In this study, we demonstrate that endothelial cells represent the major senescent population in the aged myocardium. Furthermore, we uncover a previously unrecognized role for HSPA12B as a critical regulator of age‐associated endothelial senescence and cardiac dysfunction. Specifically, HSPA12B preserves endothelial homeostasis by preventing age‐related persistent activation of the cGAS–STING pathway through regulation of ERAD‐mediated STING degradation.

Endothelial dysfunction is widely recognized as an early and prominent contributor to the development of age‐related cardiovascular diseases (Gevaert et al. 2017; Hemanthakumar et al. 2021; Minamino et al. 2002; Ungvari et al. 2018). In this study, we observed pronounced endothelial senescence phenotypes in aged hearts and established that endothelial cells constitute the predominant senescent population in the aging heart. This finding was further supported by our reanalysis of publicly available scRNA‐seq datasets from aged human cardiac tissues. These results highlight the heightened susceptibility of endothelial cells to senescence with advancing age. Given the essential role of functional endothelial cells in maintaining vascular integrity and organ homeostasis, targeting endothelial senescence may offer a promising therapeutic strategy to mitigate cardiovascular aging and improve clinical outcomes in elderly patients.

HSPA12B is predominantly expressed in endothelial cells and has previously been shown to promote angiogenesis and maintain vascular homeostasis (Han et al. 2003; Li et al. 2013; Steagall et al. 2006; Tu et al. 2020). In this study, we demonstrate that HSPA12B expression significantly declines with aging, and that endothelial‐specific deficiency of HSPA12B accelerates the development of age‐related cardiomyopathy, characterized by endothelial senescence, fibrotic remodeling, persistent inflammation, and functional decline as early as 16 months of age. These severe aging phenotypes, which are typically observed only in WT mice at 24 months or older, suggest that HSPA12B functions as a critical safeguard of endothelial integrity and cardiac health during aging.

Mechanistically, we identify HSPA12B as a novel regulator of cGAS–STING signaling. Loss of HSPA12B exacerbated cGAS–STING pathway activation, whereas HSPA12B overexpression suppressed age‐related cGAS–STING signaling. The cGAS–STING pathway is increasingly implicated in age‐related diseases, where it drives endothelial senescence, promotes inflammaging, and contributes to immunosenescence (Gulen et al. 2023; Yu et al. 2022; Zhao et al. 2023; Zhong et al. 2022). Consistent with these roles, we found that pharmacological inhibition of STING significantly reduced endothelial senescence both in vitro and in vivo. Moreover, STING inhibition using C‐176 improved cardiac function and attenuated endothelial senescence in both aged wild‐type and HSPA12B‐deficient mice. These findings further support the therapeutic potential of targeting the cGAS–STING pathway to mitigate vascular aging and preserve cardiac function.

Interestingly, while STING protein levels were markedly increased in aged endothelial cells, STING mRNA levels remained unchanged, suggesting a post‐translational regulatory mechanism. We therefore investigated whether HSPA12B modulates STING degradation. Previous studies have shown that STING is regulated through endoplasmic reticulum–associated degradation (ERAD), a process mediated by SEL1L and HRD1, two essential ERAD components (Ji et al. 2023). HRD1 functions as an ER‐resident E3 ubiquitin ligase that tags misfolded proteins for proteasomal degradation, while SEL1L acts as its adaptor protein (Doroudgar et al. 2015; Ji et al. 2023; Lin et al. 2024). We found that HSPA12B promotes the expression of both SEL1L and HRD1, whereas HSPA12B deficiency impaired their expression. Moreover, genetic knockdown of SEL1L or pharmacological inhibition of ERAD with LS‐102 abolished the protective effects of HSPA12B on STING degradation and endothelial senescence, highlighting the functional importance of the HSPA12B–ERAD axis in maintaining endothelial homeostasis.

Importantly, we identified XBP1 as a key upstream regulator linking HSPA12B to ERAD activation. XBP1 is a transcription factor critical for the expression of ERAD‐related genes (Kaneko et al. 2007; Yamamoto et al. 2008). HSPA12B physically interacted with XBP1 and promoted its nuclear translocation, thereby enhancing the transcription of SEL1L and HRD1. Inhibition of XBP1 abolished the ability of HSPA12B to facilitate ERAD‐mediated STING degradation and suppress endothelial senescence, whereas pharmacological activation of XBP1 with IXA4 recapitulated the protective effects of HSPA12B.

Collectively, this study defines a novel mechanism by which HSPA12B preserves vascular integrity during aging by facilitating ERAD‐dependent STING degradation through XBP1 activation. HSPA12B thus serves as a critical regulator of endothelial senescence and emerges as a potential therapeutic target for preventing age‐related cardiovascular disease.

## 4 Materials and Methods

### 4.1 Animals and Housing

Wild‐type C57BL/6 mice were obtained from Jackson Laboratory and housed at the Institutional Animal Care and Use Facility at East Tennessee State University. Endothelial cell specific HSPA12B knockout mice (eHSPA12B^−/−^) were generated by crossing HSPA12B^fl/fl^ with Tie2‐Cre (JAX, Stock: 008863). Male mice aged 3–5 months were categorized as young, while aged mice (22–24 months old) were bred in our laboratory and obtained from the National Institute on Aging (NIA). All animal care and experimental procedures were approved by the ETSU Committee on Animal Care and Use and adhered to NIH guidelines to ensure humane treatment. Mice were euthanized by isoflurane overdose followed by cervical dislocation. Hearts were excised immediately, rinsed in cold PBS, and transversely sectioned at the mid‐ventricular level to ensure consistent orientation across samples for histological and molecular analyses.

### 4.2 Immunofluorescence, Confocal Imaging, and Analysis

Both wild‐type (WT) and endothelial‐specific HSPA12B knockout (eHSPA12B^−/−^) mice, including young and aged cohorts, were anesthetized, and heart tissues were harvested. Tissues were fixed in 4% paraformaldehyde (PFA) for 12 h at 4°C, washed three times with cold PBS, and then immersed in 30% sucrose overnight for cryoprotection. Samples were then embedded in OCT compound and sectioned at 10 μm thickness using a Leica CM3050S cryostat. For in vitro studies, HUVECs were cultured on chamber slides, washed with PBS, and fixed in 2% PFA for 20 min at room temperature. Both cryosections and HUVECs were washed three times with PBS, permeabilized with 0.5% Triton X‐100 in PBS for 20 min at room temperature, and then blocked with 10% goat serum for 1 h. Samples were incubated overnight at 4°C with the following primary antibodies: anti‐CD31 (endothelial marker, R&D, Cat #AF3628, 1:100), anti‐p16 (Cell Signaling Technology, Cat #80772S, 1:100), anti‐p21 (Cell Signaling Technology, Cat #2947S, 1:100), anti‐dsDNA (Novus, Cat #4565, 1:100), anti‐TOM20 (mitochondrial marker, Proteintech, Cat# 66777‐1‐Ig, 1:100), and anti‐STING (ABclonal, Cat #A21051, 1:100). Following primary incubation, sections and cells were washed once with PBST and twice with PBS, then incubated with appropriate Alexa Fluor–conjugated secondary antibodies for 1 h at room temperature. After three final PBST washes, samples were mounted with DAPI‐containing fluorescence mounting medium (Thermo Fisher Scientific, Cat #P36981) to stain nuclei. Confocal images were captured using a Leica TCS SP8 microscope and processed using ImageJ software (NIH) for quantitative and qualitative analysis.

### 4.3 Cell Culture and Treatment

Human umbilical vein endothelial cells (HUVECs) were obtained from the American Type Culture Collection (ATCC, PCS‐100‐013) and cultured in Vascular Cell Basal Medium (ATCC, PCS‐100‐030) supplemented with the Endothelial Cell Growth Kit–VEGF (ATCC, PCS‐100‐041). Cells were maintained at 37°C in a humidified incubator with 5% CO₂. HUVECs at passages below P6 were considered young, while those at passage P12 or higher were classified as aged. For treatment experiments, HUVECs were exposed to the following stimuli and inhibitors, either alone or in combination as indicated: Ad‐GFP (Abm, Cat: A000541, MOI: 10), Ad‐HSPA12B (Abm, Cat: A123995, MOI: 10), C‐176 (STING inhibitor, MCE, Cat: HY‐112906, 2.5 μM), LS‐102 (HRD1 inhibitor, MCE, Cat: HY‐135844, 10 μM), MKC8866 (XBP1 inhibitor, MCE, Cat: HY‐104040, 10 μM), IXA4 (MCE, Cat: HY‐139214, 10 μM), siRNA‐control (QIAGEN, Cat: 1022076, 40 nM), siRNA‐HSPA12B (QIAGEN, Cat: SI04165637, 40 nM), and siRNA‐SEL1L (QIAGEN, Cat: SI02664494, 40 nM). As well as serum from young or aged wild‐type mice (50 μL/mL). The serum used for cell treatments was collected from young (3–5 months) and aged (22–24 months) WT mice during tissue collection, following cardiac functional assessment by echocardiography. Aged mice exhibited cardiac aging phenotypes, including endothelial cell senescence, fibrosis, and cardiac dysfunction. For each treatment, serum from 6 to 10 mice within the same group was pooled to ensure consistency. Treatments were administered for the indicated durations to evaluate changes in signaling pathways and expression of senescence‐associated markers.

### 4.4 SA‐β‐Galactosidase Staining of Cultured Cells and Frozen Sections

Senescence‐associated β‐galactosidase (SA‐β‐Gal) activity was assessed using commercial staining kits (Cell Signaling Technology, Cat# 9860S) following the manufacturers' protocols.

### 4.5 Immunoprecipitation and Western Blot Analysis

For immunoprecipitation (IP) assays, HUVECs were harvested, washed with PBS, and lysed on ice for 15 min in lysis buffer containing [1% NP‐40, 150 mM NaCl, 25 mM Tris–HCl pH 7.5, protease/phosphatase inhibitors]. Cell lysates were pre‐cleared with Protein G Magnetic Beads for 1 h at 4°C to remove nonspecific binding proteins. Following centrifugation to remove the beads, the cleared lysates were incubated overnight at 4°C with the indicated primary antibodies. Immune complexes were captured by incubation with fresh Protein G Magnetic Beads for 1 h at 4°C and collected by centrifugation. The immunoprecipitants were washed three times (5 min each) with wash buffer and separated by SDS‐PAGE for further analysis.

For Western blotting, protein was extracted from heart tissues, and in vitro cultured cells were performed using RIPA lysis buffer (Thermo Fisher Scientific, Cat: 89900) supplemented with protease and phosphatase inhibitors (Sigma‐Aldrich, Cat: PPC2020). Protein concentrations were measured using a BCA protein assay kit (Thermo Fisher Scientific, Cat: 23223). Equal amounts of protein were separated by electrophoresis on Bis‐Tris protein gels and transferred onto nitrocellulose membranes. Membranes were blocked with 5% BSA in TBS containing 0.5% Tween‐20 (TBST) for 1 h at room temperature, followed by incubation with specific primary antibodies overnight at 4°C. After washing with TBST, membranes were incubated with HRP‐conjugated secondary antibodies (Cell Signaling Technology, Cat: 7074) for 1 h at room temperature, followed by additional washes with TBST to remove unbound antibodies. Protein bands were visualized using the SuperSignal West Femto Maximum Sensitivity Substrate (Thermo Fisher Scientific, Cat: 34096), optimized for detecting low‐abundance targets, and SuperSignal West Pico PLUS Chemiluminescent Substrate (Thermo Fisher Scientific, Cat: 34580) for higher‐abundance targets. Imaging was conducted with the G:Box Chemi gel documentation system (GeneSys Version: 1.8.10.0), and band intensities were quantified using Image J software.

### 4.6 Antibodies

The following primary and secondary antibodies were used in this study: Anti‐p21 (Cat: EPR18021), anti‐p16INK4a (Cat: EPR20418) were purchased from Abcam. Anti‐CD31 (Cat: 50274) was purchased from Biosciences. Anti‐STING (Cat: 13647S), anti‐p‐STING (Cat: 72971S), anti‐TBK1 (Cat: 3504S), anti‐p‐TBK1 (Cat: 5483S), anti‐IRF3 (Cat: 4302S), anti‐p‐IRF3 (Cat: 4947S), anti‐p21 (Cat: 2947S) were purchased from Cell Signaling Technology. Anti‐p21 (Cat: A22460), anti‐p16 (Cat: A23882), anti‐A‐p53 (Cat: A19836), anti‐SEL1L (Cat: A26877), anti‐HRD1 (Cat: A2605) were purchased from Abclonal. Anti‐TOM20 (Cat: 66777–1‐Ig), anti‐XBP1S (Cat: 24168–1‐AP) were purchased from Proteintech. All primary antibodies were validated for species cross‐reactivity and used at a dilution of 1:100 for immunofluorescence or 1:1000 for Western blotting.

Alexa Fluor 488 donkey anti‐rabbit IgG (H + L) (Cat: A‐21206), Alexa Fluor 488 donkey anti‐mouse IgG (H + L) (Cat: A‐10680), Alexa Fluor 488 donkey anti‐rat IgG (H + L) (Cat: A‐21208), and Alexa Fluor 594 donkey anti‐rabbit IgG (H + L) (Cat: SA5‐10080) were purchased from Invitrogen. HRP‐conjugated goat anti‐rabbit IgG (H + L) (Cat: 7074) and HRP‐conjugated goat anti‐mouse IgG (H + L) (Cat: 7076) were purchased from Cell Signaling Technology. All secondary antibodies were validated for species cross‐reactivity and used at a dilution of 1:500 for immunofluorescence or 1:5000 for Western blotting.

### 4.7 Quantitative RT‐PCR Analysis

Total RNA was isolated from mouse heart tissues and cultured cells using RNAzol RT reagent (Molecular Research Center, Cat: RN 190) according to the manufacturer's protocol. cDNA was synthesized from 0.1 to 1 μg of total RNA using the High‐Capacity cDNA Reverse Transcription Kit (Thermo Fisher Scientific, Cat: 4368814). Quantitative real‐time PCR (qRT‐PCR) was performed using SYBR Select Master Mix on a Bio‐Rad CFX96 real‐time PCR detection system. The following PCR primers were used: Human genes: *CDKN1A* (forward, 5′‐TGTCCGTCAGAACCCATGC‐3′; reverse, 5′‐AAAGTCGAAGTTCCATCGCTC‐3′), *CDKN2A* (forward, 5′‐GGAGGCCGATCCAGGTCAT‐3′; reverse, 5′‐ATGGTTACTGCCTCTGGTGC‐3′), *IFNA1* (forward, 5′‐GCCTCGCCCTTTGCTTTACT‐3′; reverse, 5′‐CTGTGGGTCTCAGGGAGATCA‐3′), *IFNB1* (forward, 5′‐ATGACCAACAAGTGTCTCCTCC‐3′; reverse, 5′‐GGAATCCAAGCAAGTTGTAGCTC‐3′), *CXCL2* (forward, 5′‐AGATCAATGTGACGGCAGGG‐3′; reverse, 5′‐TCTCTGCTCTAACACAGAGGGA‐3′), *CXCL1* (forward, 5′‐TGCTGCCACTAATGCTGATGT‐3′; reverse, 5′‐CTCAGGAACCAATCTTTGCACT‐3′), *IL1A* (forward, 5′‐TGGTAGTAGCAACCAACGGGA‐3′; reverse, 5′‐ACTTTGATTGAGGGCGTCATTC‐3′), *STING1* (forward, 5′‐GGCCCGGATTCGAACTTACA‐3′; reverse, 5′‐TCATCTGCAGGTTCCGCTG‐3′), *SYVN1* (forward, 5′‐GCTCACGCCTACTACCTCAAA‐3′; reverse, 5′‐GCCAGACAAGTCTCTGTGACG‐3′), *SEL1L* (forward, 5′‐AAACCAGCTTTGACCGCCAT‐3′; reverse, 5′‐GTCATAGGTTGTAGCACACCAC‐3′), *XBP1* (forward, 5′‐CCCTCCAGAACATCTCCCCAT‐3′; reverse, 5′‐ACATGACTGGGTCCAAGTTGT‐3′). Mouse genes: *Cdkn1a* (forward, 5′‐CCTGGTGATGTCCGACCTG‐3′; reverse, 5′‐CCATGAGCGCATCGCAATC‐3′), *Cdkn2a* (forward, 5′‐CGCAGGTTCTTGGTCACTGT‐3′; reverse, 5′‐TGTTCACGAAAGCCAGAGCG‐3′), *Cxcl2* (forward, 5′‐ATTCTGTGACCATCCCCTCAT‐3′; reverse, 5′‐TGTATGTGCCTCTGAACCCAC‐3′), *Il1a* (forward, 5′‐CGAAGACTACAGTTCTGCCATT‐3′; reverse, 5′‐GACGTTTCAGAGGTTCTCAGAG‐3′). Primer specificity was confirmed by melting curve analysis showing single distinct peaks.

### 4.8 Mitochondrial Isolation and mtDNA Release Assay

Mitochondria were isolated using the Cell Mitochondria Isolation Kit (Thermo Fisher Scientific, Cat: 89874) according to the manufacturer's instructions. DNA was extracted from fractionated cytosolic fractions or isolated mitochondria using the DNeasy Blood and Tissue Kit (Qiagen, Cat: 69504) following the manufacturer's instructions. Mitochondrial DNA (mtDNA) levels were quantified by qRT‐PCR using specific primers for Cytochrome c oxidase I (forward: 5′‐GCCCCAGATATAGCATTCCC‐3′, reverse: 5′‐GTTCATCCTGTTCCTGCTCC‐3′). Results were normalized to 18S rRNA levels (forward: 5′‐TAGAGGGACAAGTGGCGTTC‐3′, reverse: 5′‐CGCTGAGCCAGTCAGTGT‐3′) to account for variation in sample input (Li et al. 2025).

### 4.9 Statistical Analysis

All experiments were performed at least three independent times and representative data are shown. Statistical analysis between two groups was performed by a two‐tailed Student's *t*‐test, while one‐way ANOVA with Tukey's post hoc test was used for multiple comparisons. A *p*‐value less than 0.05 was considered statistically significant. Data are expressed as mean ± SD. Analyses were performed using GraphPad Prism 8.4.3 software.

## Author Contributions

T.L. designed and performed experiments, analyzed data, interpreted results, and wrote the original manuscript. P.Z., J.A., F.T., J.W., and C.G. performed experiments and contributed to data analysis and interpretation. J.A., X.Z., L.L., S.D., K.S., D.L.W., and C.L. assisted with experimental design and data interpretation, reviewed, and revised the manuscript. X.W. designed and supervised the project and wrote the manuscript. All authors reviewed and approved the final manuscript.

## Ethics Statement

All animal studies were approved by the Animal Care and Use Committee at East Tennessee State University and were conducted in accordance with institutional guidelines and relevant regulations.

## Conflicts of Interest

The authors declare no conflicts of interest.

## Supporting information


**Figure S1:** (A‐B) Western blot analysis and quantification of senescence markers p16, p21, and acetylated p53 in heart tissues from young (4–6 months) and aged (20–24 months) mice (*n* = 6 mice/group). (C) qRT‐PCR analysis of senescence markers and senescence‐associated secretory phenotype (SASP)‐associated genes (CXCL9, IL‐1A, TNFA) in heart tissue from young and aged mice (*n* = 6 mice/group). (D‐E) Immunofluorescent staining and quantification of α‐smooth muscle actin (α‐SMA) in hearts from young and aged mice. Scale bars, 100 μm. (*n* = 6 mice/group). (F) Immunofluorescent co‐staining and quantification for p16 (red) and the endothelial marker CD31 (green) in heart sections from young (4–6 months) or aged (20–24 months) mice (*n* = 6 mice/group). Scale bars, 50 μm. Data are presented as the mean ± SD. *p < 0.05, **p < 0.01, ***p < 0.001.
**Figure S2:** (A) Immunofluorescent co‐staining of CD31 with HSPA12B in heart tissues from WT and eHSPA12B^−^/^−^ mice (*n* = 6/group). Scale bars, 50 μm. (B) Representative Picrosirius Red staining of heart sections from 16‐month‐old eHSPA12B^−^/^−^ and WT mice. Scale bar, 100 μm. (C‐D) Representative confocal immunofluorescent images of SA‐β‐gal and p16 positive cells in the indicated groups (*n* = 6/group).
**Figure S3:** (A‐B) Representative images and quantification of β‐gal–positive cells in HUVECs transfected with si‐Control or si‐HSPA12B and treated with serum from young or aged mice for 24 h. Scale bars, 100 μm. (C) qRT‐PCR analysis of p16 and p21 mRNA expression in HUVECs transfected with si‐Control or si‐HSPA12B and cultured at young (P6) or aged (P14) passages. (*n* = 6/group). (D‐E) Representative images and quantification of β‐gal–positive cells in the indicated groups (*n* = 6/group). (F) Immunoblot analysis of HSPA12B in HUVECs transfected with Ad‐GFP or Ad‐HSPA12B. (G‐J) Representative images and quantification of β‐gal–positive cells in the indicated groups (*n* = 6/group). (K) Immunoblot analysis of senescence markers in HUVECs transfected with Ad‐GFP or Ad‐HSPA12B and treated with serum from young or aged mice for 24 h (*n* = 4/group). (L) qRT‐PCR analysis of p16 and p21 mRNA expression in the indicated groups (*n* = 6/group). Data are presented as the mean ± SD. *p < 0.05, **p < 0.01, ***p < 0.001.
**Figure S4:** (A) Quantification of STING pathway components in cardiac endothelial cells isolated from young and aged mice (*n* = 3 mice/group). (B‐C) Western blot analysis and quantification of STING pathway components in heart tissue from young and aged mice (*n* = 3 mice/group). (D) Quantification of STING pathway components in young (P < 6) and aged (P12–16) HUVECs (*n* = 3/group). (E) Western blot analysis of cGAS expression in young versus aged HUVECs. (*n* = 3/group). (F) Western blot analysis of cGAS expression in young (P < 6) and aged (P12–16) HUVECs with or without si‐Control or si‐HSPA12B transfection. (G) Western blot analysis of cGAS expression in young (P < 6) and aged (P12–16) HUVECs with or without Ad‐HSPA12B transfection. (H‐I) Western blot analysis of STING pathway components, p21, and p16 in HUVECs treated with vehicle control, the STING inhibitor C‐176, si‐Control, or si‐HSPA12B. Data are presented as the mean ± SD. *p < 0.05, **p < 0.01, ***p < 0.001.
**Figure S5:** (A) Representative confocal immunofluorescent staining of phosphorylated STING, TBK1, and IRF3 (p‐STING, p‐TBK1, p‐IRF3, red) co‐stained with the endothelial marker CD31 (green) in heart sections from young, aged, and aged + C‐176–treated WT and eHSPA12B^−^/^−^ mice. Nuclei counterstained with DAPI (blue). Scale bars, 50 μm.
**Figure S6:** (A‐B) Immunofluorescent co‐staining and quantification of CD31 and p21 in heart tissues from C‐176– and vehicle‐treated aged WT and eHSPA12B^−^/^−^ mice. Scale bars, 50 μm (*n* = 6/group). (C‐D) Representative β‐galactosidase staining and quantification of HUVECs in the indicated groups (*n* = 6/group). Scale bars, 100 μm. (E‐F) Representative imaged and quantification of p21‐positive endothelial cells in the indicated groups (*n* = 6/group). (G) qRT‐PCR analysis of SASP‐associated cytokines and chemokines in young and aged HUVECs treated with vehicle or C‐176 (*n* = 6/group). Data are presented as the mean ± SD. *p < 0.05, **p < 0.01, ***p < 0.001.
**Figure S7:** (A‐B) Immunofluorescent staining and quantification of p21‐positive endothelial cells in aged HUVECs were transfected with siRNA targeting SEL1L or control siRNA, followed by adenoviral overexpression of HSPA12B (*n* = 6/group). Scale bars, 50 μm. (C‐D) Immunofluorescent staining and quantification of p21‐positive endothelial cells in the indicated groups (*n* = 6/group). Scale bars, 50 μm. Data are presented as the mean ± SD. *p < 0.05, **p < 0.01, ***p < 0.001.
**Figure S8:** (A) qPCR analysis of senescence markers, SEL1L and HRD1 mRNA levels in aged HUVECs were transfected with siRNA targeting SEL1L or control siRNA, followed by adenoviral overexpression of HSPA12B (Ad‐HSPA12B) or GFP (Ad‐GFP) (*n* = 6/group). (B) qRT‐PCR analysis of p16 and p21 mRNA expression in aged HUVECs were treated with the HRD1 inhibitor LS‐102 to inhibit ERAD, followed by Ad‐HSPA12B or Ad‐GFP transduction (*n* = 6/group). (C) Immunofluorescent staining of XBP1s in young and aged endothelial cells treated with IXA4. Scale bars, 50 μm. (D) qRT‐PCR analysis of XBP‐1S in the indicated HUVEC groups (*n* = 6/group). (E) Quantification of p21‐positive endothelial cells in the indicated groups (n = 6/group). (F) qRT‐PCR analysis of SEL1L and HRD1 mRNA levels in young and aged endothelial cells treated with IXA4 (*n* = 6/group). (G) qRT‐PCR analysis of senescence markers in aged HUVECs were treated with the MKC8866, followed by adenoviral overexpression of HSPA12B (Ad‐HSPA12B) or GFP (Ad‐GFP) (*n* = 6/group). (H) Immunofluorescent staining of p21 in the indicated groups. Scale bars, 50 μm. (I) Representative images of β‐gal–positive cells in aged HUVECs were treated with the MKC8866, followed by adenoviral overexpression of HSPA12B (Ad‐HSPA12B) or control vector (Ad‐GFP). Scale bars, 100 μm. Data are presented as the mean ± SD. *p < 0.05, **p < 0.01, ***p < 0.001.

## Data Availability

All data generated or analyzed during this study is available from the corresponding author upon reasonable request.

## References

[acel70260-bib-0001] Bloom, S. I. , M. T. Islam , L. A. Lesniewski , and A. J. Donato . 2023. “Mechanisms and Consequences of Endothelial Cell Senescence.” Nature Reviews Cardiology 20: 38–51. 10.1038/s41569-022-00739-0. 35853997 PMC10026597

[acel70260-bib-0002] Booth, L. K. , R. E. Redgrave , S. Tual‐Chalot , I. Spyridopoulos , H. M. Phillips , and G. D. Richardson . 2023. “Heart Disease and Ageing: The Roles of Senescence, Mitochondria, and Telomerase in Cardiovascular Disease.” Sub‐Cellular Biochemistry 103: 45–78. 10.1007/978-3-031-26576-1_4. 37120464

[acel70260-bib-0003] Brandes, R. P. , I. Fleming , and R. Busse . 2005. “Endothelial Aging.” Cardiovascular Research 66, no. 2: 286–294. 10.1016/j.cardiores.2004.12.027. 15820197

[acel70260-bib-0004] Cabral‐Miranda, F. , G. Tamburini , G. Martinez , et al. 2022. “Unfolded Protein Response IRE1/XBP1 Signaling Is Required for Healthy Mammalian Brain Aging.” EMBO Journal 41, no. 22: e111952. 10.15252/embj.2022111952. 36314651 PMC9670206

[acel70260-bib-0005] Chen, Y. , C. Yang , Y. Miao , et al. 2025. “Macrophage STING Signaling Promotes Fibrosis in Benign Airway Stenosis via an IL6‐STAT3 Pathway.” Nature Communications 16, no. 1: 289. 10.1038/s41467-024-55170-5. PMC11698984 39753529

[acel70260-bib-0006] Childs, B. G. , H. Li , and J. M. van Deursen . 2018. “Senescent Cells: A Therapeutic Target for Cardiovascular Disease.” Journal of Clinical Investigation 128, no. 4: 1217–1228. 10.1172/JCI95146. 29608141 PMC5873883

[acel70260-bib-0007] Culley, M. K. , and S. Y. Chan . 2022. “Endothelial Senescence: A New Age in Pulmonary Hypertension.” Circulation Research 130, no. 6: 928–941. 10.1161/CIRCRESAHA.121.319815. 35298304 PMC8943914

[acel70260-bib-0008] Doroudgar, S. , M. Völkers , D. J. Thuerauf , et al. 2015. “Hrd1 and ER‐Associated Protein Degradation, ERAD, Are Critical Elements of the Adaptive ER Stress Response in Cardiac Myocytes.” Circulation Research 117, no. 6: 536–546. 10.1161/CIRCRESAHA.115.306993. 26137860 PMC4670262

[acel70260-bib-0009] Dvorkin, S. , S. Cambier , H. E. Volkman , and D. B. Stetson . 2024. “New Frontiers in the cGAS‐STING Intracellular DNA‐Sensing Pathway.” Immunity 57, no. 4: 718–730. 10.1016/j.immuni.2024.02.019. 38599167 PMC11013568

[acel70260-bib-0010] Evangelou, K. , P. V. S. Vasileiou , A. Papaspyropoulos , et al. 2023. “Cellular Senescence and Cardiovascular Diseases: Moving to the ‘Heart’ of the Problem.” Physiological Reviews 103, no. 1: 609–647. 10.1152/physrev.00007.2022. 36049114

[acel70260-bib-0011] Fujita, H. , N. Yagishita , S. Aratani , et al. 2015. “The E3 Ligase Synoviolin Controls Body Weight and Mitochondrial Biogenesis Through Negative Regulation of PGC‐1β.” EMBO Journal 34, no. 8: 1042–1055. 10.15252/embj.201489897. 25698262 PMC4406651

[acel70260-bib-0012] Gevaert, A. B. , H. Shakeri , A. J. Leloup , et al. 2017. “Endothelial Senescence Contributes to Heart Failure With Preserved Ejection Fraction in an Aging Mouse Model.” Circulation. Heart Failure 10, no. 6: e003806. 10.1161/CIRCHEARTFAILURE.116.003806. 28611124

[acel70260-bib-0013] Glück, S. , B. Guey , M. F. Gulen , et al. 2017. “Innate Immune Sensing of Cytosolic Chromatin Fragments Through cGAS Promotes Senescence.” Nature Cell Biology 19, no. 9: 1061–1070. 10.1038/ncb3586. 28759028 PMC5826565

[acel70260-bib-0014] Gulen, M. F. , N. Samson , A. Keller , et al. 2023. “cGAS‐STING Drives Ageing‐Related Inflammation and Neurodegeneration.” Nature 620, no. 7973: 374–380. 10.1038/s41586-023-06373-1. 37532932 PMC10412454

[acel70260-bib-0015] Guo, Q. , X. Chen , J. Chen , et al. 2021. “STING Promotes Senescence, Apoptosis, and Extracellular Matrix Degradation in Osteoarthritis via the NF‐κB Signaling Pathway.” Cell Death & Disease 12, no. 1: 13. 10.1038/s41419-020-03341-9. 33414452 PMC7791051

[acel70260-bib-0016] Han, Z. , Q. A. Truong , S. Park , and J. L. Breslow . 2003. “Two Hsp70 Family Members Expressed in Atherosclerotic Lesions.” Proceedings of the National Academy of Sciences of the United States of America 100, no. 3: 1256–1261. 10.1073/pnas.252764399. 12552099 PMC298760

[acel70260-bib-0017] He, B. , H. Yu , S. Liu , et al. 2022. “Mitochondrial Cristae Architecture Protects Against mtDNA Release and Inflammation.” Cell Reports 41, no. 10: 111774. 10.1016/j.celrep.2022.111774. 36476853

[acel70260-bib-0018] Hemanthakumar, K. A. , S. Fang , A. Anisimov , M. I. Mäyränpää , E. Mervaala , and R. Kivelä . 2021. “Cardiovascular Disease Risk Factors Induce Mesenchymal Features and Senescence in Mouse Cardiac Endothelial Cells.” eLife 10: e62678. 10.7554/eLife.62678. 33661096 PMC8043751

[acel70260-bib-0019] Ji, Y. , Y. Luo , Y. Wu , et al. 2023. “SEL1L‐HRD1 Endoplasmic Reticulum‐Associated Degradation Controls STING‐Mediated Innate Immunity by Limiting the Size of the Activable STING Pool.” Nature Cell Biology 25, no. 5: 726–739. 10.1038/s41556-023-01138-4. 37142791 PMC10185471

[acel70260-bib-0020] Jiménez‐Loygorri, J. I. , B. Villarejo‐Zori , Á. Viedma‐Poyatos , et al. 2024. “Mitophagy Curtails Cytosolic mtDNA‐Dependent Activation of cGAS/STING Inflammation During Aging.” Nature Communications 15, no. 1: 830. 10.1038/s41467-024-45044-1. PMC10821893 38280852

[acel70260-bib-0021] Kaneko, M. , S. Yasui , Y. Niinuma , et al. 2007. “A Different Pathway in the Endoplasmic Reticulum Stress‐Induced Expression of Human HRD1 and SEL1 Genes.” FEBS Letters 581, no. 28: 5355–5360. 10.1016/j.febslet.2007.10.033. 17967421

[acel70260-bib-0022] Kanemaru, K. , J. Cranley , D. Muraro , et al. 2025. “Author Correction: Spatially Resolved Multiomics of Human Cardiac Niches.” Nature 640, no. 8058: E4. 10.1038/s41586-025-08886-3. 40113895 PMC11981930

[acel70260-bib-0023] Li, H. , M. H. Hastings , J. Rhee , L. E. Trager , J. D. Roh , and A. Rosenzweig . 2020. “Targeting Age‐Related Pathways in Heart Failure.” Circulation Research 126, no. 4: 533–551. 10.1161/CIRCRESAHA.119.315889. 32078451 PMC7041880

[acel70260-bib-0024] Li, J. , Y. Zhang , C. Li , et al. 2013. “HSPA12B Attenuates Cardiac Dysfunction and Remodelling After Myocardial Infarction Through an eNOS‐Dependent Mechanism.” Cardiovascular Research 99, no. 4: 674–684. 10.1093/cvr/cvt139. 23729663

[acel70260-bib-0025] Li, T. , J. Adams , P. Zhu , et al. 2025. “The Role of Heme in Sepsis Induced Kupffer Cell PANoptosis and Senescence.” Cell Death & Disease 16, no. 1: 284. 10.1038/s41419-025-07637-6. 40221420 PMC11993645

[acel70260-bib-0026] Lin, L. L. , H. H. Wang , B. Pederson , et al. 2024. “SEL1L‐HRD1 Interaction Is Required to Form a Functional HRD1 ERAD Complex.” Nature Communications 15, no. 1: 1440. 10.1038/s41467-024-45633-0. PMC10873344 38365914

[acel70260-bib-0027] Litviňuková, M. , C. Talavera‐López , H. Maatz , et al. 2020. “Cells of the Adult Human Heart.” Nature 588, no. 7838: 466–472. 10.1038/s41586-020-2797-4. 32971526 PMC7681775

[acel70260-bib-0028] Liu, H. , S. Ghosh , T. Vaidya , et al. 2023. “Activated cGAS/STING Signaling Elicits Endothelial Cell Senescence in Early Diabetic Retinopathy.” JCI Insight 8, no. 12: e168945. 10.1172/jci.insight.168945. 37345657 PMC10371250

[acel70260-bib-0029] Marino, F. , M. Scalise , N. Salerno , et al. 2022. “Diabetes‐Induced Cellular Senescence and Senescence‐Associated Secretory Phenotype Impair Cardiac Regeneration and Function Independently of Age.” Diabetes 71, no. 5: 1081–1098. 10.2337/db21-0536. 35108360 PMC9490451

[acel70260-bib-0030] Martin, S. S. , A. W. Aday , N. B. Allen , et al. 2025. “2025 Heart Disease and Stroke Statistics: A Report of US and Global Data From the American Heart Association.” Circulation 151, no. 8: e41–e660. 10.1161/CIR.0000000000001303. 39866113 PMC12256702

[acel70260-bib-0031] Minamino, T. , H. Miyauchi , T. Yoshida , Y. Ishida , H. Yoshida , and I. Komuro . 2002. “Endothelial Cell Senescence in Human Atherosclerosis: Role of Telomere in Endothelial Dysfunction.” Circulation 105, no. 13: 1541–1544. 10.1161/01.cir.0000013836.85741.17. 11927518

[acel70260-bib-0032] Reiterer, M. , and C. M. Branco . 2020. “Endothelial Cells and Organ Function: Applications and Implications of Understanding Unique and Reciprocal Remodelling.” FEBS Journal 287, no. 6: 1088–1100. 10.1111/febs.15143. 31736207 PMC7155104

[acel70260-bib-0033] Sayed, N. , Y. Huang , K. Nguyen , et al. 2021. “An Inflammatory Aging Clock (iAge) Based on Deep Learning Tracks Multimorbidity, Immunosenescence, Frailty and Cardiovascular Aging.” Nature Aging 1: 598–615. 10.1038/s43587-021-00082-y. 34888528 PMC8654267

[acel70260-bib-0034] Sheng, X. , H. Z. Nenseth , S. Qu , et al. 2019. “IRE1α‐XBP1s Pathway Promotes Prostate Cancer by Activating c‐MYC Signaling.” Nature Communications 10, no. 1: 323. 10.1038/s41467-018-08152-3. PMC6345973 30679434

[acel70260-bib-0035] Sidney, S. , A. S. Go , M. G. Jaffe , M. D. Solomon , A. P. Ambrosy , and J. S. Rana . 2019. “Association Between Aging of the US Population and Heart Disease Mortality From 2011 to 2017.” JAMA Cardiology 4, no. 12: 1280–1286. 10.1001/jamacardio.2019.4187. 31663094 PMC6822092

[acel70260-bib-0036] Steagall, R. J. , A. E. Rusiñol , Q. A. Truong , and Z. Han . 2006. “HSPA12B Is Predominantly Expressed in Endothelial Cells and Required for Angiogenesis.” Arteriosclerosis, Thrombosis, and Vascular Biology 26, no. 9: 2012–2018. 10.1161/01.ATV.0000235720.61091.c7. 16825593

[acel70260-bib-0037] Sun, Z. , and V. Hornung . 2022. “cGAS‐STING signaling.” Current Biology 32, no. 13: R730–R734. 10.1016/j.cub.2022.05.027. 35820380

[acel70260-bib-0038] Tu, F. , X. Wang , X. Zhang , et al. 2020. “Novel Role of Endothelial Derived Exosomal HSPA12B in Regulating Macrophage Inflammatory Responses in Polymicrobial Sepsis.” Frontiers in Immunology 11: 825. 10.3389/fimmu.2020.00825. 32457753 PMC7221167

[acel70260-bib-0039] Ungvari, Z. , S. Tarantini , T. Kiss , et al. 2018. “Endothelial Dysfunction and Angiogenesis Impairment in the Ageing Vasculature.” Nature Reviews Cardiology 15, no. 9: 555–565. 10.1038/s41569-018-0030-z. 29795441 PMC6612360

[acel70260-bib-0040] United States Census Bureau . 2023. 2023 National Population Projections Tables: Main Series. United States Census Bureau.

[acel70260-bib-0041] Yamamoto, K. , N. Suzuki , T. Wada , et al. 2008. “Human HRD1 Promoter Carries a Functional Unfolded Protein Response Element to Which XBP1 but Not ATF6 Directly Binds.” Journal of Biochemistry 144, no. 4: 477–486. 10.1093/jb/mvn091. 18664523 PMC2755579

[acel70260-bib-0042] Yin, K. , D. Patten , S. Gough , et al. 2022. “Senescence‐Induced Endothelial Phenotypes Underpin Immune‐Mediated Senescence Surveillance.” Genes & Development 36, no. 9–10: 533–549. 10.1101/gad.349585.122. 35618311 PMC9186388

[acel70260-bib-0043] Yu, H. , K. Liao , Y. Hu , et al. 2022. “Role of the cGAS‐STING Pathway in Aging‐Related Endothelial Dysfunction.” Aging and Disease 13, no. 6: 1901–1918. 10.14336/AD.2022.0316. 36465181 PMC9662267

[acel70260-bib-0044] Zhang, X. , X. Wang , M. Fan , et al. 2020. “Endothelial HSPA12B Exerts Protection Against Sepsis‐Induced Severe Cardiomyopathy via Suppression of Adhesion Molecule Expression by miR‐126.” Frontiers in Immunology 11: 566. 10.3389/fimmu.2020.00566. 32411123 PMC7201039

[acel70260-bib-0045] Zhao, Y. , M. Simon , A. Seluanov , and V. Gorbunova . 2023. “DNA Damage and Repair in Age‐Related Inflammation.” Nature Reviews. Immunology 23, no. 2: 75–89. 10.1038/s41577-022-00751-y. PMC10106081 35831609

[acel70260-bib-0046] Zhong, W. , Z. Rao , J. Xu , et al. 2022. “Defective Mitophagy in Aged Macrophages Promotes Mitochondrial DNA Cytosolic Leakage to Activate STING Signaling During Liver Sterile Inflammation.” Aging Cell 21, no. 6: e13622. 10.1111/acel.13622. 35599014 PMC9197407

