# Supplementary material for: HSPA12B Protects Against Age‐Related Endothelial Cell Senescence by Regulating STING Degradation
Source: Aging Cell. 2025 Oct 8;24(12):e70260. doi: 10.1111/acel.70260 (PMC12686555; doi:10.1111/acel.70260)
Supplement: Supplementary file 1 — Figure S1: (A‐B) Western blot analysis and quantification of senescence markers p16, p21, and acetylated p53 in heart tissues from young (4–6 months) and aged (20–24 months) mice (n = 6 mice/group). (C) qRT‐PCR analysis of senescence markers and senescence‐associated secretory phenotype (SASP)‐associated genes (CXCL9, IL‐1A, TNFA) in heart tissue from young and aged mice (n = 6 mice/group). (D‐E) Immunofluorescent staining and quantification of α‐smooth muscle actin (α‐SMA) in hearts from young and aged mice. Scale bars, 100 μm. (n = 6 mice/group). (F) Immunofluorescent co‐staining and quantification for p16 (red) and the endothelial marker CD31 (green) in heart sections from young (4–6 months) or aged (20–24 months) mice (n = 6 mice/group). Scale bars, 50 μm. Data are presented as the mean ± SD. *p < 0.05, **p < 0.01, ***p < 0.001. Figure S2: (A) Immunofluorescent co‐staining of CD31 with HSPA12B in heart tissues from WT and eHSPA12B−/− mice (n = 6/group). Scale bars, 50 μm. (B) Representative Picrosirius Red staining of heart sections from 16‐month‐old eHSPA12B−/− and WT mice. Scale bar, 100 μm. (C‐D) Representative confocal immunofluorescent images of SA‐β‐gal and p16 positive cells in the indicated groups (n = 6/group). Figure S3: (A‐B) Representative images and quantification of β‐gal–positive cells in HUVECs transfected with si‐Control or si‐HSPA12B and treated with serum from young or aged mice for 24 h. Scale bars, 100 μm. (C) qRT‐PCR analysis of p16 and p21 mRNA expression in HUVECs transfected with si‐Control or si‐HSPA12B and cultured at young (P6) or aged (P14) passages. (n = 6/group). (D‐E) Representative images and quantification of β‐gal–positive cells in the indicated groups (n = 6/group). (F) Immunoblot analysis of HSPA12B in HUVECs transfected with Ad‐GFP or Ad‐HSPA12B. (G‐J) Representative images and quantification of β‐gal–positive cells in the indicated groups (n = 6/group). (K) Immunoblot analysis of senescence markers in HUVECs transfected wit [file ACEL-24-e70260-s001.docx]

**HSPA12B protects against age related endothelial cell senescence by regulating ERAD mediated STING degradation**

Tingting Li, Peilin Zhu, Joseph Adams, Fei Tu, Jialing Wang, Chloe Garbe, Suman Dalal, Krishna Singh, Xiaojin Zhang, Li Liu, David L Williams, Chuanfu Li, Xiaohui Wang

**Table of contents**

Supplementary figures...............................................................................................................2

**Figure S1: (A-B)** Western blot analysis and quantification of senescence markers p16, p21, and acetylated p53 in heart tissues from young (4–6 months) and aged (20–24 months) mice (n=6 mice/group). **(C)** qRT-PCR analysis of senescence markers and senescence-associated secretory phenotype (SASP)-associated genes (CXCL9, IL-1A, TNFA) in heart tissue from young and aged mice (n=6 mice/group). **(D-E)** Immunofluorescent staining and quantification of α-smooth muscle actin (α-SMA) in hearts from young and aged mice. Scale bars, 100 μm. (n=6 mice/group). **(F)** Immunofluorescent co-staining and quantification for p16 (red) and the endothelial marker CD31 (green) in heart sections from young (4–6 months) or aged (20–24 months) mice (n=6 mice/group). Scale bars, 50 μm. Data are presented as the mean ± SD. *P < 0.05, **P < 0.01, ***P < 0.001.

**Figure S2: (A)** Immunofluorescent co-staining of CD31 with HSPA12B in heart tissues from WT and eHSPA12B⁻/⁻ mice (n=6/group). Scale bars, 50 μm. **(B)** Representative Picrosirius Red staining of heart sections from 16-month-old eHSPA12B⁻/⁻ and WT mice. Scale bar, 100 μm. **(C-D)** Representative confocal immunofluorescent images of SA-β-gal and p21 positive cells in the indicated groups (n=6/group).

 **Figure S3: (A-B)** Representative images and quantification of β-gal–positive cells in HUVECs transfected with si-Control or si-HSPA12B and treated with serum from young or aged mice for 24 hours. Scale bars, 100 μm. **(C)** qRT-PCR analysis of p16 and p21 mRNA expression in HUVECs transfected with si-Control or si-HSPA12B and cultured at young (P6) or aged (P14) passages. (n=6/group). **(D-E)** Representative images and quantification of β-gal–positive cells in the indicated groups (n=6/group). **(F)** Immunoblot analysis of HSPA12B in HUVECs transfected with Ad-GFP or Ad-HSPA12B. **(G-J)** Representative images and quantification of β-gal–positive cells in the indicated groups (n=6/group). **(K)** Immunoblot analysis of senescence markers in HUVECs transfected with Ad-GFP or Ad-HSPA12B and treated with serum from young or aged mice for 24 hours (n=4/group). **(L)** qRT-PCR analysis of p16 and p21 mRNA expression in the indicated groups (n=6/group). Data are presented as the mean ± SD. *P < 0.05, **P < 0.01, ***P < 0.001.

**Figure S4: (A)** Quantification of STING pathway components in cardiac endothelial cells isolated from young and aged mice (n=3 mice/group). **(B-C)** Western blot analysis and quantification of STING pathway components in heart tissue from young and aged mice (n=3 mice/group). **(D)** Quantification of STING pathway components in young (P<6) and aged (P12–16) HUVECs (n=3/group). **(E)** Western blot analysis of cGAS expression in young versus aged HUVECs. (n=3/group). **(F)** Western blot analysis of cGAS expression in young (P<6) and aged (P12–16) HUVECs with or without si-Control or si-HSPA12B transfection. **(G)** Western blot analysis of cGAS expression in young (P<6) and aged (P12–16) HUVECs with or without Ad-HSPA12B transfection. **(H-I)** Western blot analysis of STING pathway components, p21, and p16 in HUVECs treated with vehicle control, the STING inhibitor C-176, si-Control, or si-HSPA12B. Data are presented as the mean ± SD. *P < 0.05, **P < 0.01, ***P < 0.001.

**Figure S5: (A)** Representative confocal immunofluorescent staining of phosphorylated STING, TBK1, and IRF3 (p-STING, p-TBK1, p-IRF3, red) co-stained with the endothelial marker CD31 (green) in in heart sections from young, aged, and aged + C-176–treated WT and eHSPA12B⁻/⁻ mice. Nuclei counterstained with DAPI (blue). Scale bars, 50 μm.

**Figure S6: (A-B)** Immunofluorescent co-staining and quantification of CD31 and p21 in heart tissues from C-176– and vehicle-treated aged WT and eHSPA12B⁻/⁻ mice. Scale bars, 50 μm (n=6/group). **(C-D)** Representative β-galactosidase staining and quantification of aged HUVECs in the indicated groups (n=6/group). Scale bars, 100 μm. **(E-F)** Representative imaged and quantification of p21-positive endothelial cells in the indicated groups (n=6/group). **(G)** qRT-PCR analysis of SASP-associated cytokines and chemokines in young and aged HUVECs treated with vehicle or C-176 (n = 6/group). Data are presented as the mean ± SD. *P < 0.05, **P < 0.01, ***P < 0.001.

**Figure S7: (A-B)** Immunofluorescent staining and quantification of p21-positive endothelial cells in aged HUVECs were transfected with siRNA targeting SEL1L or control siRNA, followed by adenoviral overexpression of HSPA12B (n=6/group). Scale bars, 50 μm. **(C-D)** Immunofluorescent staining and quantification of p21-positive endothelial cells in the indicated groups (n=6/group). Scale bars, 50 μm. Data are presented as the mean ± SD. *P < 0.05, **P < 0.01, ***P < 0.001.

**Figure S8: (A)** qPCR analysis of senescence markers, SEL1L and HRD1 mRNA levels in aged HUVECs were transfected with siRNA targeting SEL1L or control siRNA, followed by adenoviral overexpression of HSPA12B (Ad-HSPA12B) or GFP (Ad-GFP) (n=6/group). **(B)** qRT-PCR analysis of p16 and p21 mRNA expression in aged HUVECs were treated with the HRD1 inhibitor LS-102 to inhibit ERAD, followed by Ad-HSPA12B or Ad-GFP transduction (n=6/group). **(C)** Immunofluorescent staining of XBP1s in young and aged endothelial cells treated with IXA4. Scale bars, 50 μm. **(D)** qRT-PCR analysis of XBP-1S in the indicated HUVEC groups (n=6/group). **(E)** Quantification of p21-positive endothelial cells in the indicated groups (n=6/group). **(F)** qRT-PCR analysis of SEL1L and HRD1 mRNA levels in young and aged endothelial cells treated with IXA4 (n=6/group). **(G)** qRT-PCR analysis of senescence markers in aged HUVECs were treated with the MKC8866, followed by adenoviral overexpression of HSPA12B (Ad-HSPA12B) or GFP (Ad-GFP) (n=6/group). **(H)** Immunofluorescent staining of p21 in the indicated groups. Scale bars, 50 μm. **(I)** Representative images of β-gal–positive cells in aged HUVECs were treated with the MKC8866, followed by adenoviral overexpression of HSPA12B (Ad-HSPA12B) or control vector (Ad-GFP). Scale bars, 100 μm. Data are presented as the mean ± SD. *P < 0.05, **P < 0.01, ***P < 0.001.
